# Novelties in Hybrid Zones: Crossroads between Population Genomic and Ecological Approaches

**DOI:** 10.1371/journal.pone.0000357

**Published:** 2007-04-04

**Authors:** Caroline Costedoat, Nicolas Pech, Rémi Chappaz, André Gilles

**Affiliations:** Evolution Genome Environment (EA 3781 EGEE), Université de Provence, Marseille, France; Ecole Normale Supérieure de Lyon, France

## Abstract

**Background:**

Interspecific hybridization is widespread, occurring in a taxonomically diverse array of species. The Cyprinidae family, which displays more than 30% hybridization, is a good candidate for studies of processes underlying isolation and speciation, such as genetic exchange between previously isolated lineages**.** This is particularly relevant in the case of recent hybridization between an invasive species, *Chondrostoma nasus nasus* (from Eastern Europe), and *C. toxostoma toxostoma* (a threatened species endemic to southern France), in which bidirectional introgressive hybridization has been demonstrated.

**Methodology/Principal Findings:**

We studied 128 specimens from reference populations and 1495 hybrid zone specimens (two years of sampling and four stations), using five molecular markers (one mitochondrial gene, four nuclear introns), morphology (meristic and plastic characters) and life history traits (weight, size, coefficient of condition, sex, age, shoaling). We identified 65 hybrid combinations and visualized spatial and temporal changes in composition. The direction of mitochondrial introgression was density-dependent in favor of the rarer species and we demonstrate that the sexual selection hypothesis is a preponderant explanation in the asymmetry of introgression. Despite genomic evolution in the hybrid zone, convergence was observed for body shape and coefficient of condition, indicating changes in foraging behavior with respect to reference populations, reflecting strong environmental pressure.

**Conclusions/Significance:**

The complex rules of hybrid zone dynamics are established very early in the contact zone. We propose “inheritance from the rare species” as a new evolutionary hypothesis for animal models. The endemic species was not assimilated by the invasive species. Survival rates for this species were highest in the middle of the river (the warmest part) due to a trade-off between food availability and fecundity. The environment-independent hybrid combination may result from nuclear-mitochondrial interactions involving the *Tpi1b* gene or a gene linked to this gene (Chromosome 16). This genomic region is also responsible for shoaling behavior in *Danio rerio* and is a promising zone for studies of changes in population dynamics and advances in integrated studies of hybrid zones.

## Introduction

Hybridization between species and subsequent backcrossing may lead to the transfer of genes between species. This introgression of new genetic information provides new variability and is a major factor in subsequent evolution. However, the genetic assimilation of a rare species by more common congeners [e.g. in 1], [2], [3] has important implications for conservation. Genetic models have indicated that this process may occur extremely quickly, and many examples have been described in both plants [4], [5] and animals [3], [6]. Hybridization is therefore now considered a serious threat to endangered species. Wilson [7] reported that 38% of the 292 North American fishes listed as rare, threatened, or vulnerable in the Red Data Book published by the International Union for the Conservation of Nature and Natural Resources were threatened by hybridization. Scribner *et al.* [8] reviewed 158 articles covering 19 fish families from North America, Europe and Asia. In “natural” conditions (i.e. excluding fish farming conditions), species from the Cyprinidae included more hybrid pairs than any other family, with more than 30% hybrids, whereas hybridization rates were 8% in the Salmonidae and only 3% in the Cichlidae. The preponderance of this phenomenon in this family, its worldwide distribution and the inclusion in this family of a model species (*Danio rerio*) render this family particularly interesting for studies of hybridization phenomena in an evolutionary context.

*Chondrostoma t. toxostoma* is a threatened, protected endemic cyprinid species from southern France. Part of its distribution range has been colonized by the invasive *Chondrostoma n. nasus*, from Eastern Europe. This colonization has made hybridization between these two species possible, particularly in the Rhone Basin. In previous studies [9], [10], we reported bidirectional introgression between the two *Chondrostoma* species in the River Durance. The structuring of the hybrid zone was strongly linked to progressive urbanization and an increase in human activities along this river, with the construction of various dams preventing fish from swimming upstream or downstream. No continuum was found between the species inhabiting the tributaries (*C. t. toxostoma*) upstream and the species inhabiting the river (*C. n. nasus*) downstream. There was therefore no hybrid gradient based on the ecological preferences of the species, as would have been expected based on the route followed by *C. n. nasus* during the colonization process. These findings conflict with those for two other well known cyprinid models: a gradient hybrid zone has been reported between *Barbus barbus* and *Barbus meridionalis* in the Lergue (Europe) [11] and asymmetric introgression in two *Luxilus* species (America) [12] has been accounted for by historic effects (refuge populations and dispersal route).

Dams delimited four main stations displaying independent evolution as it was almost impossible for the fish to move from one station to another.

We evaluated the contributions of genome and environment (Eco-Geno context) to spatial and temporal changes in introgressive hybridization, to enable us to decipher hybridization phenomena and the rules governing them. We used species-specific molecular diagnosis profiles to quantify levels of hybridization and introgression in the two species. Changes in size may be associated with changes in shape, and may have functional significance [13]. However, few studies have considered changes in ecological characters or morphological analyses of hybridization, and most such studies have focused on a limited number of hybrid classes [e.g. in 14]. We studied each of the two *Chondrostoma* species in allopatric and parapatric populations (reference values) and then focused on the hybrid zone. The River Durance was divided into four sampling stations, from upstream to downstream, which were sampled in 2001 and in 2002. All 1623 specimens were identified on the basis of molecular markers (mtDNA *cytochrome b* and four nuclear introns), and life history traits, such as sex, age, weight, size, coefficient of condition, shoaling behavior and morphology (meristic and plastic characters), were analyzed.

## Results

### Specimen identification and dynamics of the hybrid zone

**Figure 1:**
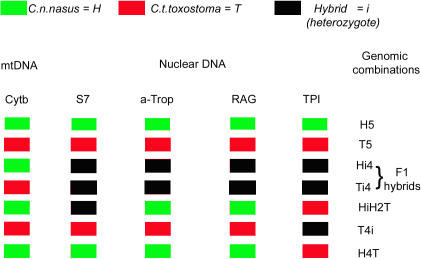
IDENTIFICATION CODE. Some examples of genomic combinations based on the alleles for each of the five molecular markers (1 mitochondrial gene and 4 nuclear introns).

All 1623 specimens were identified based on the five molecular markers (*cytochrome b* plus the four introns). The allele dynamics for each marker are presented in Dataset S1, figures S1, S2, S3, S4; Tables S1, S2. We developed an identification code based on the results obtained for each marker (figure 1), defining 162 combinations: the two parental combinations plus 160 hybrid categories. As expected, all specimens from allopatric and parapatric populations were identified as “pure” specimens with our markers. In the hybrid zone, we observed 67 of the 162 possible combinations: the two parental combinations (H5 and T5) and 65 hybrid combinations.

We tested the null hypothesis of Hardy-Weinberg equilibrium, using chi-squared goodness-of-fit tests, with Benjamini-Hochberg correction for multiple comparisons. We classified the genomic combinations into three major groups: combinations overrepresented under the null hypothesis of Hardy-Weinberg equilibrium, those underrepresented and those observed in the expected proportions.

Forty-one of the 65 hybrid combinations observed (considering the entire data set) presented *nasus* mtDNA and 24, *toxostoma* mtDNA (significant difference: chi-square = 6.73; df = 1; *p* = 0.009). Both types of F1 hybrid were present, but at different frequencies (Table 1): 31 Hi4 specimens versus 5 Ti4 specimens (chi-square = 18.78; df = 1; *p* = 1.5×10^−5^). We then investigated the temporal and spatial dynamics of evolution in the hybrid zone, by studying the data sets for each year (2001 and 2002) and sampling station (Buech, Manosque, Pertuis and Cavaillon) separately (Table 1).

**Table 1:** OBSERVED AND EXPECTED EFFECTIVE SIZE OF EACH GENOMIC COMBINATIONS PRESENT IN THE HYBRID ZONE

	2001		2002		BUECH		MANOSQUE		PERTUIS		CAVAILLON	
Combi.	Obs.	Exp.	Obs.	Exp.	Obs.	Exp.	Obs.	Exp.	Obs.	Exp.	Obs.	Exp.
H5	**82**	0.019	**169**	0.059	**134**	1.075	**26**	1.386e-04	**4**	3.507e-08	**87**	35.822
H2i2T	1		1		1		0		1		0	
H2i3	3		1		3		0		**1**	7.067e-04	0	
H2iH2	0		1		1		0		0		0	
H2iHT	0		1		0		0		**1**	4.132e-04	0	
H2iT2	1		0		0		0		1		0	
H2iTi	1		0		0		1		0		0	
H2TH2	1		0		0		0		**1**	3.775e-06	0	
H3i2	1		1		0		1		**1**	3.406e-05	0	
H3iH	**5**	0.069	2		5		**2**	1.144e-03	0		*0*	9.215
H3iT	1		1		1		0		**1**	4.057e-04	0	
H3T2	1		1		2		0		0		0	
H3TH	0		1		0		**1**	2.362e-03	0		0	
H4i	**2**	0.109	**6**	0.264	5		**2**	1.184e-03	**1**	1.671e-06	0	
H4T	**25**	0.156	**17**	0.292	**28**	2.034	**8**	2.531e-03	**3**	1.991e-05	3	
Hi2H2	1		0		0		0		**1**	1.486e-05	0	
Hi2Hi	0		1		1		0		0		0	
Hi2HT	1		0		0		1		0		0	
Hi3H	4		1		4		0		0		1	
Hi3T	10		8		5		1		**12**	1.719e-12	0	
Hi4	10		21		20		**10**	7.232e-01	1		0	
HiH2i	1		1		0		1		**1**	3.414e-05	0	
HiH2T	3		0		3		0		0		0	
HiH3	0		**3**	0.209	1		**1**	1.162e-03	0		*1*	11.703
HiHi2	0		1		1		0		0		0	
HiHiH	1		1		0		1		**1**	1.149e-05	0	
HiHiT	1		0		0		0		1		0	
HiTHi	0		1		0		1		0		0	
HiTi2	0		3		3		0		0		0	
HiTiT	*1*	9.466	3		1		0		3		0	
HT3i	1		*0*	8.496	0		1		0		0	
HT4	5		6		1		10		*0*	23.203	0	
HTH2T	**7**	0.645	0		7	1.553	0		0		0	
HTH3	**2**	0.079	0		0		**2**	2.436e-03	0		0	
HTHiH	1		0		1		0		0		0	
HTHiT	0		1		0		1		0		0	
HTHTH	0		3		0		**3**	4.153e-02	0		0	
HTHTi	0		1		0		1		0		0	
HTi2T	2		*0*	11.557	1		0		1		0	
HTi3	4		*0*	10.468	3		0		1		0	
HTiT2	*0*	8.645	*1*	10.397	0		1		0		0	
HTiTi	0		2		0		2		0		0	
T5	**246**	14.620	**431**	14.636	**147**	0.533	**243**	58.440	**280**	149.684	**7**	2.337e-07
T2HT2	5		0		0		1		1		**3**	2.101e-05
T2i2T	*1*	15.889	*0*	18.034	0		*0*	12.844	1		0	
T2i3	*2*	11.151	*1*	16.334	1		1		1		0	
T2iT2	*1*	14.466	*4*	16.224	3		*1*	26.516	*1*	28.854	0	
T3H2	1		0		0		1		0		0	
T3Hi	1		0		1		0		0		0	
T3HT	**11**	4.421	0		**8**	0.816	3		0		0	
T3iT	*2*	16.080	*2*	16.268	1		*1*	28.308	*1*	29.385	**1**	3.635e-06
T4H	4		0		2		0		0		**2**	2.696e-05
T4i	**27**	10.260	9		**5**	0.775	28		3		0	
TH4	**2**	0.032	**4**	0.093	1		**5**	5.495e-04	0		0	
THi3	0		2		0		2		0		0	
THiHi	0		1		0		1		0		0	
THT3	3		2		3		2		0		0	
THTHT	0		4		0		**4**	1.950e-01	0		0	
THTiT	1		0		1		0		0		0	
Ti2T2	*0*	14.211	*1*	18.158	1		*0*	12.649	0		0	
Ti3T	*4*	15.631	*1*	20.650	0		4		1		0	
Ti4	*1*	10.969	*4*	18.704	*0*	10.044	5		0		0	
TiH3	0		1		0		0		**1**	4.621e-06	0	
TiT2i	*0*	10.093	*1*	15.180	0		*1*	13.045	0		0	
TiT3	16		*2*	16.759	**7**	1.220	*11*	27.878	*0*	29.315	0	
TiTHT	2		1		0		3		0		0	
TiTiT	*0*	15.818	*2*	18.628	0		*2*	13.504	0		0	
Total	509		733		413		397		327		105	

We indicated only the expected effective when the difference observed-expected (under Hypothesis of Hardy-Weinberg equilibrium) was significant. Separately for each samling year and for each sampling station. Obs.: Observed value; Exp.: Expected value; Bold: overrepresented combinations; italic: underrepresented combinations.

### Alternate asymmetric mtDNA introgression

We investigated the distribution of the hybrid combinations as a function of the abundance of the parental species. The mtDNA of the hybrid combinations (particularly for the overrepresented combinations) originated in most cases from the rarer of the two “parental” species. Indeed, for the total data set, more *C. t. toxostoma* individuals (677) than *C. n. nasus* individuals (251) were observed, whereas the hybrid combinations were more likely to present *C. n. nasus* mtDNA (188 vs 126; *p*<10^−15^) (Table 2). The same trend was observed if the data for each sampling year and for each sampling station were considered separately (Table 2). We call this phenomenon “inheritance from the rare species”.

**Table 2:** DISTRIBUTION OF THE OR *TOXOSTOMA* MTDNA MODALITY.

		Parents		hybrids			hy over rep.				hy under rep.				hy expected			
mtDNA		Eff.	Clas.	Eff.	Fischer	Clas.	Eff.	Fischer	Clas.	Fischer	Eff.	Fischer	Clas.	Fischer	Eff.	Fischer	Clas.	Fischer
**Global**	N	251	1	**188**	**p<10^−10^	**41**	**98**	**p<10^−10^	**6**	p = 0.40400	17	p = 0.53000	7	**p = 0.00002	**73**	**p<10^−10^	**28**	p = 0.24400
	T	**677**	1	126		24	42		2		**47**		**11**		37		11	
**Buech**	N	134	1	**98**	**p<10^−10^	**22**	**35**	*p = 0.02100	2	p = 0.28000	0		0		**63**	**p<10^−10^	**20**	p = 0.1788
	T	**147**	1	34		12	20		3		0		0		14		9	
**Manosque**	N	26	1	52	**p<10^−10^	**21**	**29**	**p<10^−10^	**8**	p = 0.12600	0	p = 0.20700	0	*p = 0.03090	23	**p<10^−10^	**13**	p = 0.59300
	T	**243**	1	**76**		18	9		2		**16**		**5**		**51**		11	
**Pertuis**	N	4	1	**33**	**p<10^−10^	**18**	**24**	**p<10^−10^	**11**	p = 0.13400	0	p = 0.95800	0	p = 0.11900	**9**	**p<10^−10^	**7**	p = 0.38100
	T	**280**	1	10		8	1		1		**3**		**2**		7		5	
**Cavaillon**	N	**87**	1	5	**p = 0.00035	3	0	**p = 0.00001	0	p = 0.23800	**1**	p = 0.92600	**1**	p = 0.57100	**4**	p = 0.73900	**2**	p = 0.35700
	T	7	1	**6**		3	**5**		**3**		0		0		0		0	
**2001**	N	82	1	**97**	**p<10^−10^	**28**	**41**	**p = 0.00001	**5**	p = 0.65400	1	p = 0.13200	1	*p = 0.02300	**55**	**p<10^−10^	**22**	p = 0.22700
	T	**246**	1	84		17	40		3		**12**		**6**		32		8	
**2002**	N	169	1	**91**	**p<10^−10^	**28**	**26**	**p<10^−10^	**3**	p = 0.53000	1	*p = 0.01710	1	*p = 0.00328	**64**	**p<10^−10^	**24**	p = 0.12400
	T	**431**	1	42		17	4		1		**18**		**9**		20		7	

Detailed of the “heritage of the rare” phenomenon. “Parents”: correspond to the H5 and T5, “Hybrids” : correspond to all the hybrids. Then the three last columns detailed the hybrids combination (i.e. overrepresented; underrepresented and in expected proportion). N: *C.n. nasus*; T: *C. t.toxostoma*; Eff.: effective size; N.Clas.: number of genomic classes; Fischer: results of the exact Fischer test calculated first with the effective size; then with the number of class (respective references are in grey).

### Morphology and hybridization

**Figure 2:**
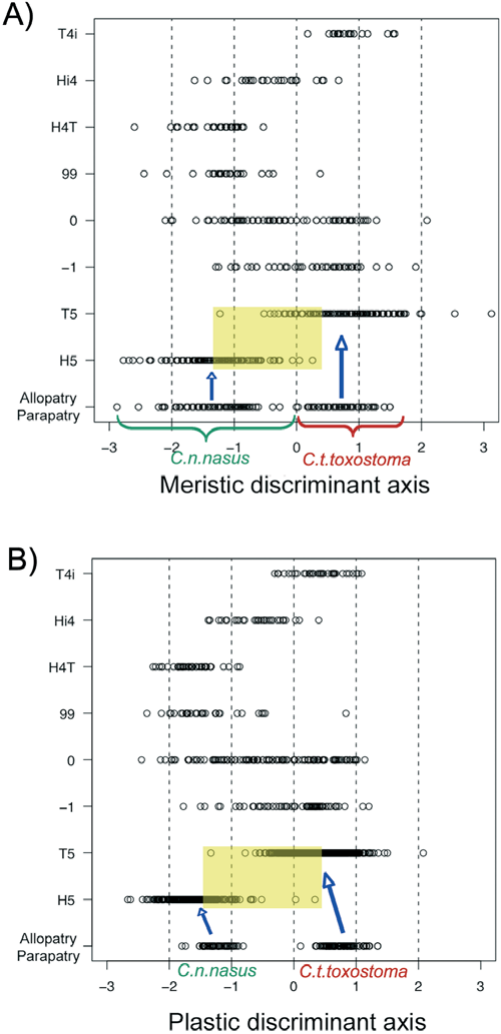
VISUALIZATION OF THE VARIANCE FOR MORPHOLOGY. Discriminant analysis based on meristic characters (x-axis) as a function of genetic class (y-axis). -1 = underrepresented combinations; 0 = combinations present in the expected proportions; 99 = overrepresented combinations other than T5, H5,T4i, Hi4 and H4T, which are represented separately (see the text for more detail). A) Meristic data set; B) Plastic data set on the global data set.

We calculated the coordinates of each hybrid zone specimen on the discriminant axis defined by the reference populations (figure 2). The morphological ranges of the H5 and T5 classes were larger than those of the two species sampled in allopatry and parapatry and, furthermore, these ranges overlapped. The morphological range of the Hi4 specimens (F1 with *nasus* mtDNA) was intermediate between those of the H5 and T5 specimens (figure 2). See Dataset S2, figures S5 and S6.

**Figure 3:**
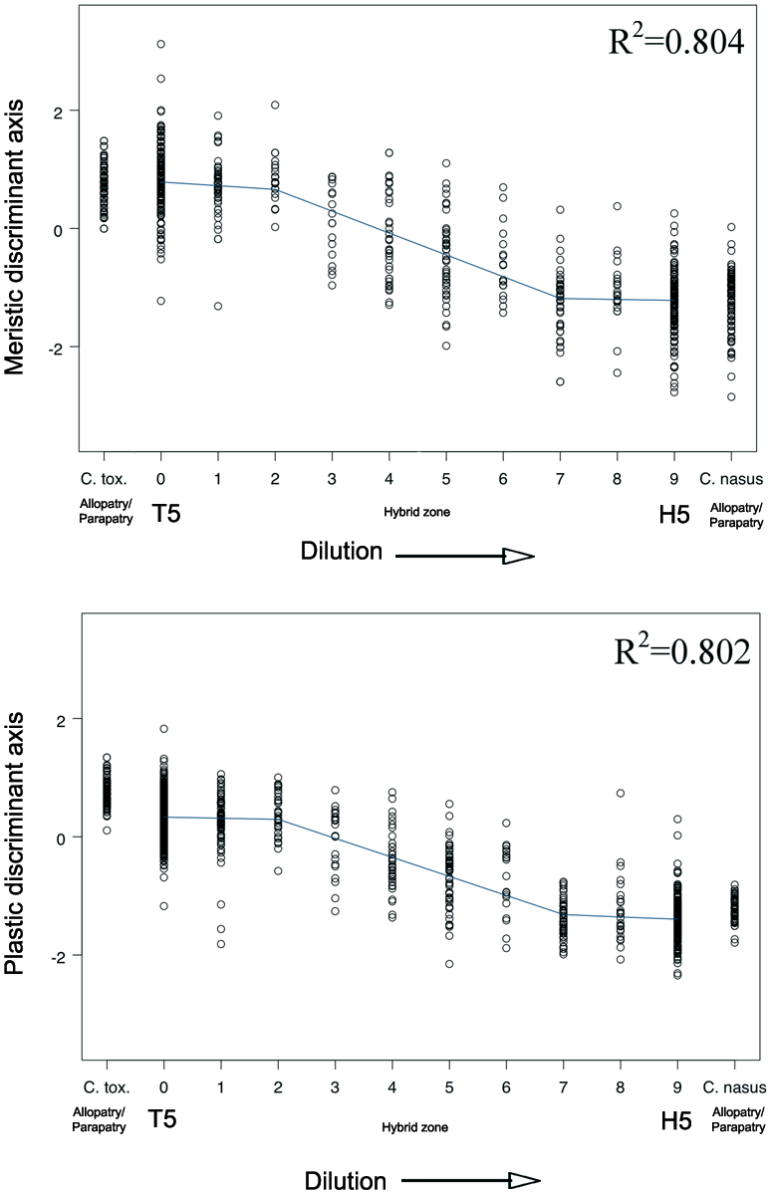
CORRELATION BETWEEN MORPHOLOGY AND GENE DILUTION. Correlation between morphology (meristic at the top and plastic at the bottom) and the degree of introgression (percentage of genes belonging to each of the two species) or dilution.

Based on plastic morphology, H5 and T5 specimens in the hybrid zone were mostly classified in hybrid classes, rather than with their respective reference populations (Table S3), illustrating the difference in morphology between individuals in the hybrid zone and reference allopatry/parapatry populations (not observed with meristic characters). We therefore assessed the correlation between gene dilution (*i.e.* the proportion of alleles from each species present in each genomic combination) and morphology. The coefficient of determination was significant for both meristic and plastic characters (R^2^ = 0.804 for meristic characters; and R^2^ = 0.802 for plastic characters, figure 3).

**Figure 4:**
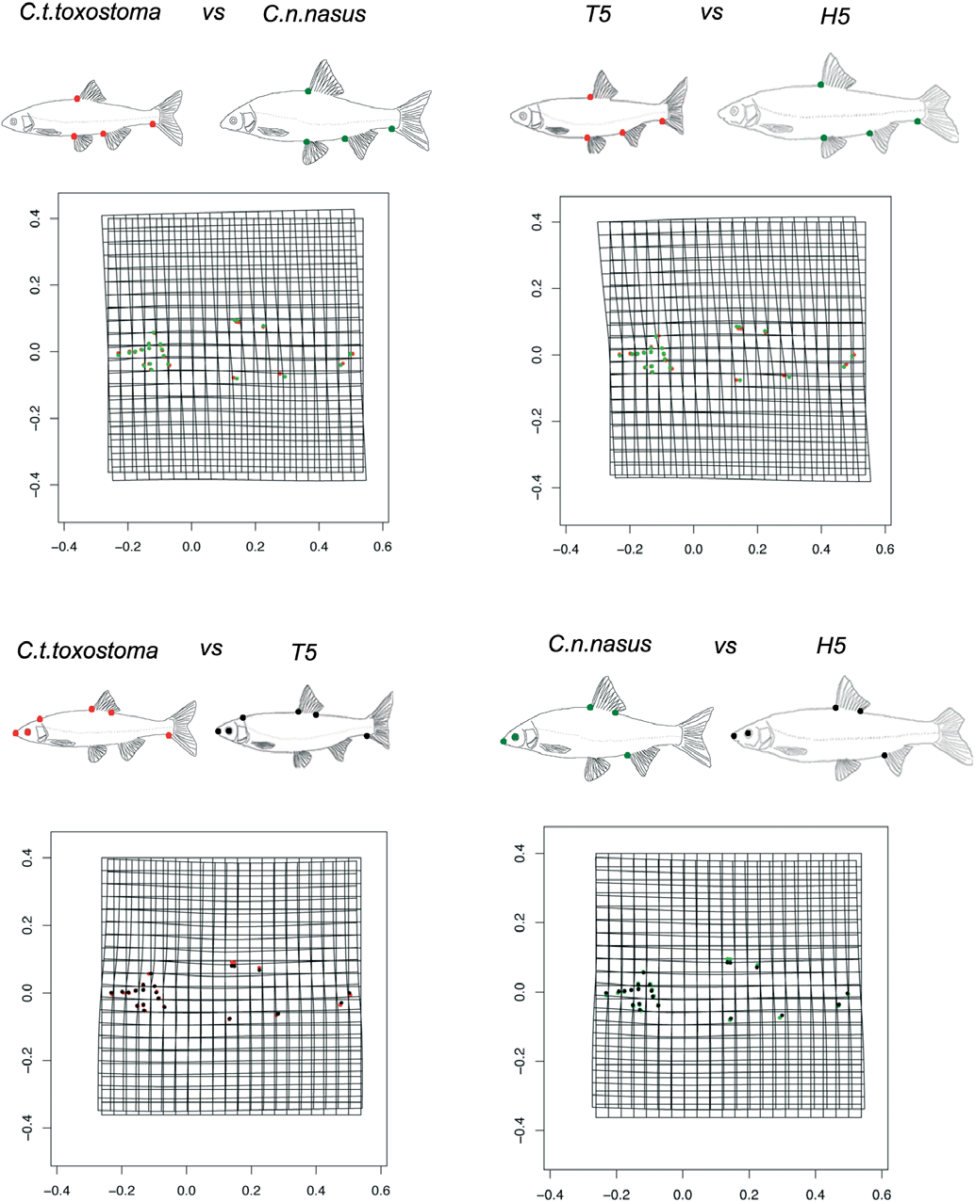
DEFORMATION GRIDS. Deformation grids for various “mean specimen” comparisons (based on the 21 landmarks). These diagrams are schematic representations of the morphology encountered in allopatric/parapatric zones or in hybrid zones. The points indicate the main deformations observed on the corresponding grid.

All genomic categories displayed a shift in body shape from those of the reference populations (figure 2). This shift corresponded to a movement towards the *nasus* type (figure 2). A similar shift was observed for individuals of all sizes and ages and from all stations (data not shown). We visualized this change, using deformation grids for different comparisons (figure 4). The main differences between the mean reference *nasus* individual and the mean H5 individual concerned snout shape and body height between the dorsal fin and the pelvic fin, which was smaller in the H5 specimen (figure 4). Surprisingly, the deformation from a reference mean *toxostoma* to a mean T5 was similar. Both the mean T5 specimen and the mean H5 specimen were more rounded than the corresponding mean reference specimens. All hybrid zone specimens presented similar body shape deformations, regardless of their genomic class. These changes resulted from an effect of the Durance river environment rather than a genomic effect (Table S4).

### Life history traits

In reference populations, the mean coefficient of condition value for *C. n. nasus* (K_C.n.nasus_ = 12.50) differed significantly from that for *C. t. toxostoma* (K_C.t.toxostoma_ = 9.84; *p*<10^−15^). However, no species effect was found in the hybrid zone (*p* = 0.16), with K_H5_ = 11.01; K_T5_ = 11.22. We can summarize this relationship as follows: K_C.n.nasus_>K_H5_ = K_T5_>K_C.t.toxostoma_

**Figure 5:**
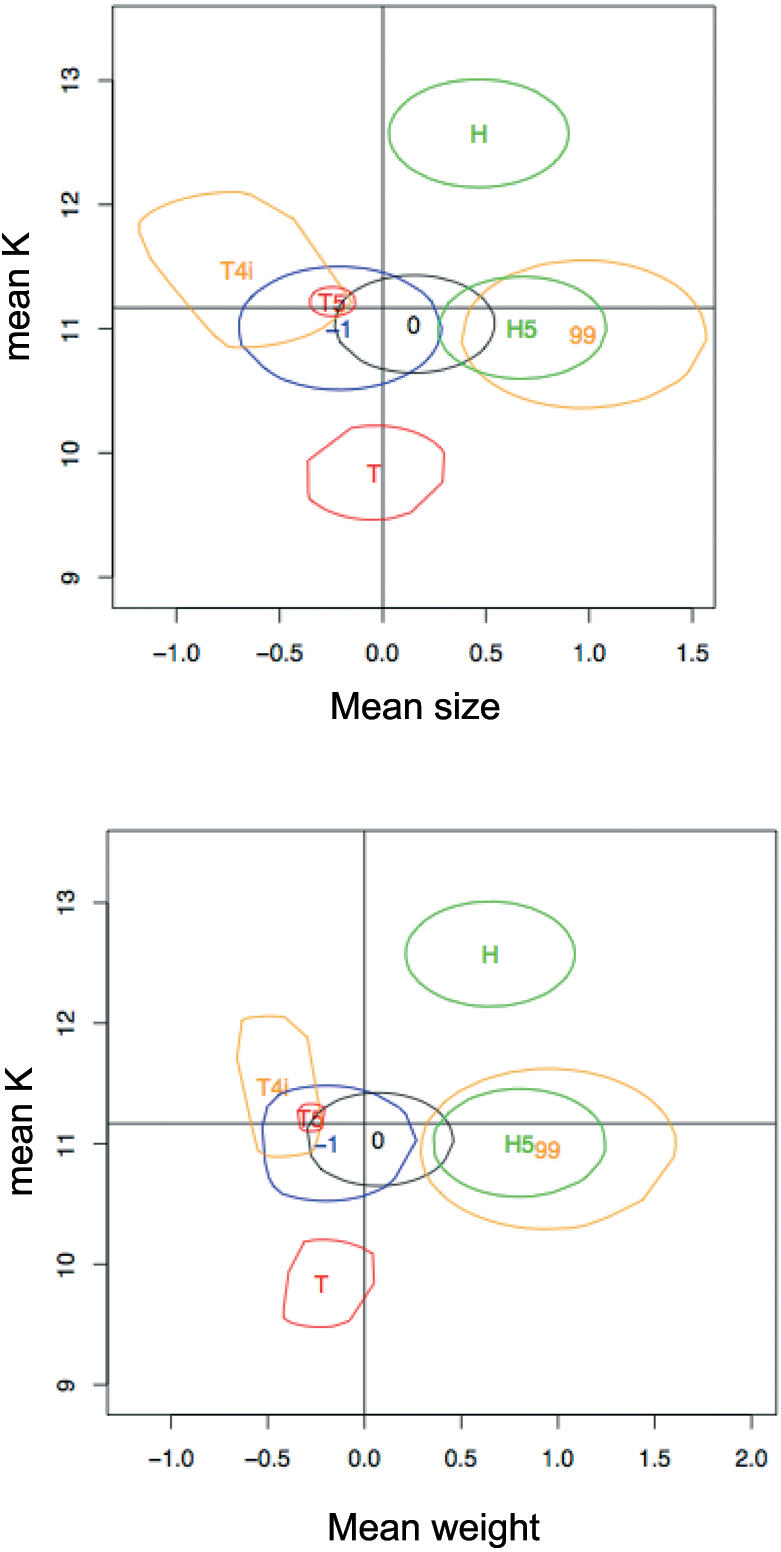
COEFFICIENT OF CONDITION AND CONFIDENCE SURFACES. Confidence surfaces for mean K-size (a) and mean K-weight (b) plots, based on non-parametric bootstrap (1000 replicates). The circles correspond to 95% of bootstrapped values. H and T correspond to *C. n. nasus* and *C. t. toxostoma*, respectively, in the reference populations. The other classes are those of the hybrid zone (T5; H5; -1 = underrepresented hybrid combinations; 0 = hybrid combinations found in the expected proportions; 99 = overrepresented hybrid combinations; T4i = the only hybrid combination for which sufficient specimens were obtained for comparison alone, without pooling). The variance of K is very significantly different (Bartlett B = 63.7; *p* = 10^−10^) between classes.

The greater K value for the reference *C. n. nasus* specimen than for H5 seems to result principally from the larger size of the H5 specimens (figure 5). Conversely, T5 specimens were smaller than *C. t. toxostoma* specimens and the difference in weight between the two was smaller than that for H5 and *C. n. nasus*. (More details in Dataset S2, figure S7, Table S5).

The oldest specimens (10 years old) were of the *C. n. nasus* species and the sex ratio in the hybrid zone was about 1 (Tables S6, S7).

### Interactions between variables

We tested potential interactions between variables, using chi-square tests (second-order interactions) and log-linear models (third-order interactions) for qualitative variables. The third-order interaction of the saturated log linear model (genomic class×station×sampling year) was significant (dev. = 72.134; p = 5×10^−10^). The main interaction results are reported Table S8.

Positive interactions were found between H5 and the two extreme stations (the Buech station upstream and the Cavaillon station downstream) and between T5 and the two intermediate stations (Manosque and Pertuis). This is consistent with the distribution of the different genomic groups not following an upstream-downstream gradient. Some hybrid combinations were positively associated with a particular station (e.g. T4i and the Manosque station), and were not present at others. These combinations may be considered to be environment-dependent. In contrast, H4T (positively associated with the Buech station) was overrepresented at three of the four stations and present in the expected proportions at the fourth. This combination may therefore be considered environment-independent.

## Discussion

### From deciphering *Chondrostoma* hybrid zone dynamics to the emergence of an evolutionary scenario (figure 6)

**Figure 6:**
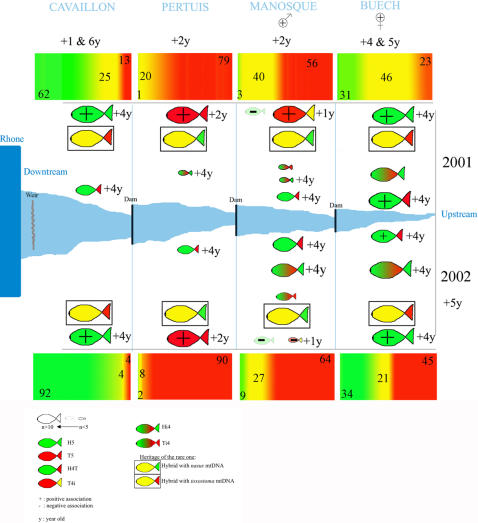
GENOMIC EVOLUTION THROUGH THE HYBRID ZONE. Global summary of the dynamics of the hybrid zone and main variable interactions (chi-square test and log-linear model) with respect to the various sampling years and stations (see the text for more detail). The percentages of the different categories are shown—from *T5* in red to *H5* in green, and “hybrids” in yellow—separately for each station and each sampling year.

Our study confirmed the bidirectionality of introgression between the *Chrondrostoma* species, via the two F1 hybrid pools identified as Hi4 and Ti4. The hybrid specimens displayed no signs of sterility, with hybrid males producing sperm during the reproductive period and hybrid females presenting normal development, with a micropyle (Stolzenberg personal communication). We identified environment-dependent and environment-independent combinations and describe here the first vertebrate hybrid zone following the “evolutionary novelty” model defined by Arnold [15], with two types of genotype variation encountered within the same species complex and in the same river. However, only one (H4T) of the 65 hybrid combinations observed was clearly environment-independent. This combination consisted of *C. t. toxostoma* alleles for the Tpi 1b intron and *C. n. nasus* alleles for all the other molecular markers. Recent studies have highlighted the importance of interactions between the mitochondrial and nuclear compartments, particularly for fitness (amount of available energy [16], [17]), in all biological models. The Tpi 1b gene product is involved in glycolysis. We therefore searched the *Danio rerio* chromosome map for other genes encoding proteins involved in glycolysis. G3PDH, like Tpi 1b, was found to be present on chromosome 16 (region displayed: 58.99-59.01 cM). We identified no other candidate genes from the *Danio rerio* map in this chromosome region, but a more precise estimate of the *Chondrostoma* genome is required to demonstrate such a direct interaction.

The hybrid zone presented a mosaic of genomic combinations evolving over space and time (figure 6). The tendency of some genomic combinations (H5, T5 and some hybrid ones) to be overrepresented may reflect assortative mating, leading to a tendency to display intraspecific shoaling in both species (as suggested by the frequent sampling of specimens of similar genomic combination and age together). Recent studies have highlighted the role of intraspecific and interspecific shoaling in the dynamics of fish populations and its consequences for individual fitness [18], [19], [20]. Such shoaling behavior cannot itself account for the pattern of evolution, but Persat (personal communication) has reported peaks in the numbers of *C. nasus nasus* in the River Rhone every four or five years that could account for dynamic changes in the hybrid zone (which tended to be confirmed by the distribution of age classes) in the two sampling years. Indeed, the structure of fish communities has great potential for rapid change in response to habitat loss and human activities modifying habitat. This is particularly true of group-spawning species (e.g. Cyprinidae) that focus their reproductive activities on specific spawning substrates, often within very restricted areas.

Our results show that environment may play a key role in determining the directionality of hybridization and the maintenance of particular hybrid combinations and we provide the first evidence of reversible directionality (or more precisely intensity) of gene flow between two species in the same river. The introgression observed was bidirectional, but the direction of mitochondrial introgression was density-dependent, favoring the rare species (figure 6). This “inheritance from the rare species” may be due to sexual selection [21], with all females fertilized but rare males having to compete directly with the males of the common species. Avise and Saunders [22] came close to formulating this hypothesis. However, they did not explain why the same argument did not apply to males and simply assumed differences in the abundances of the sexes in *Lepomis* species. Our field study (with a sex ratio of about 1) provides the first demonstration of asymmetric mtDNA introgression due to sexual selection. Indeed, we were able to test the hypothesis of “inheritance from the rare species” because we observed an alternation of gene flow between the two species in the same river, and because we knew that hybridization was contemporary, making it possible to “calibrate” each of the two species in allopatric zones. The station-dependence of the proportion of the rare species and of inheritance from the rare species indicates that this phenomenon is dependent on population dynamics.

### Asymmetric mtDNA introgression and “inheritance from the rare species”: a ubiquitous pattern

Some studies have reported cases of unequal gene exchange between species [23], [24] or of cytonuclear disequilibrium and mitochondrial capture ([25], and more recently [26]), in which the mtDNA genotypes characteristic of one species occur in a predominant nuclear background from another species. The “capture” phenomenon is observed in both plants and animals, from fish to mice [25]. Most of these studies provisionally eliminated lineage sorting from a polymorphic ancestor as an alternative explanation and documented the widespread occurrence of historical gene exchange between species. They concluded that this capture phenomenon was due either to ancient introgressive hybridization between the related species with the “mother species” outnumbered in the past, or biases in species sampling [e.g. in], [27], [Bibr pone.0000357-Wayne1] [28; 29; 30]. We list examples of hybridization in the animal kingdom in Table 3, focusing particularly on the mitochondrial DNA present in the hybrids (similar conclusions could be inferred from Y chromosome data but the fish studied here has no sexual chromosome. We therefore limited our interpretation to mitochondrial DNA and the maternal lineage). In 65% of all reported cases, the hybrids contained the mitochondrial DNA of only one of the two parental species. Not all authors have tried to explain the absence of one of the two possible mtDNA types in the hybrids observed. Others have proposed various “prezygotic” and “postzygotic” hypotheses (cf. “authors conclusion” in Table 3). Rather than focusing on these hypotheses, we considered our own biological model and the characteristics of our hybrid zone. We concluded that the “most extreme case” presented by Wirtz [21] (with some crosses of two given species giving different results in different areas) was probably not an extreme scenario and was probably more common than initially thought and should be considered in other fish and metazoan models. Indeed, recent studies have shown that there are more hybrid taxa than expected in the animal kingdom [31], and comparisons of the results obtained in different studies on different biological models [e.g. 32], [33] may lead to the identification of general processes. For example, flycatcher hybrids are less fit than their parents [34]. However, the presence of these hybrids was accounted for by particular demographic situations in which it was better for females to mate with heterospecific males rather than not to mate at all. Arnold found this conclusion “intriguing” [35]. An “intriguing” feature of our work is that our model fish, which is very different in terms of reproduction and mate choice strategies (group spawner versus strong mating preference and partner choice), seems to display similar general biological behavior. Our “inheritance of rare species” hypothesis may thus be more general, possibly accounting for the bidirectional mtDNA introgression observed by Thulin *et al*. in 1997 and 2003 [36], [37] in hare species (Table 3). However, more detailed studies are required to confirm this.

**Table:** ASYMMETRICAL INTROGRESSION IN ANIMAL KINGDOM.

Genus	Common name	Ref.	Hybrids mtDNA	Author's conclusion
**Amphibians**				
* Hyla cinerea x H. gratiosa*	Frog	[65]	*H. gratiosa*	Females of *H. gratiosa* have to ‘hop the gauntlet” of *H cinerea* males before reaching conspecific mating partners.
* Rana lessonae x R. ridibunda*	Frog	[66]	Both	Both field observations and studies of mating preference indicate that the primary hybridizations that produce *R. esculenta* are between *R. ridibunda* females and *R. lessonae* males; thereafter, *R. esculenta* lineages are usually maintained by matings of *R. esculenta* females with *R. lessonae* males
**Birds**				
* Vermivora pinus x V. chrysoptera*	Warbler	[67]	Both	The rapid pace of asymmetrical introgression may be the result of initial invasion of chrysoptera populations by pioneering female pinus and/or an unknown competitive advantage of pinus females and their daughters over chrysoptera females.
* Stercorarius pomarinus x S. skua*	Parasitic jaeger	[68]	*S. pomarinus*	Past introgressive hybridization
**Crustaceans**				
* Mytilus edulis x M. galloprovincialis*	Mussel	[69]	Both	Predominant mtDNA flow from *M. edulis* to *M. galloprovincialis*. This can be explained by the dispersal of larvae to South West England from *M. edulis* regions to the north and east, but little dispersal in the opposite directions.
**Fish**				
* Salvelinus fontinalis x S. alpinus*	Charr and trout	[70]	*S. alpinus*	Combination of both historical demographic conditions and selection for mtDNA introgression, rather than pure stochastic processes, as a more plausible mechanism which could have produced the present- day geographical distribution observed for introgressed brook char in eastern Quebec.
* Salvelinus namaycush x S. alpinus*	*Charr and trout*	[71]	*S. alpinus*	Although *S. alpinus* is not found in Lac des Chasseurs or its drainage, it is probable that the species colonized the area soon after deglaciation, followed later by lake trout. An alternate hypothesis is that hybrids may have been favoured under periglacial conditions, due to selective advantages in having the *S. alpinus* mitochondrial type (cf their discussion).
* Lepomis macrochirus x L. microlophus*	Sunfish	[22]	Both	The data support the idea that hybridizations preferentially take place between parental species differing greatly in abundance. Other crosses with asymmetrical results: *Lepomis macrochirus* x *L. cyanelus* = *L. cyanellus*
* Micropterus punctatus x M. dolomieui*	Black bass	[72]	Both (but…)	Predominance of *M. dolomieui* mtDNA. In cytonuclear genetic examinations of natural hybridization involving other species of Centrarchidae, Avise and Saunders (1984) noted a strong tendency for locally rare species to provide the female parent in interspecific crosses. This same pattern appears to hold at the present time for the spotted/ smallmouth complex.
* Notropis cornutus x N. chrysocephalus*	Shiner	[73]	Both (but…)	The proportion depending on the abundance of the parental species. The rarer species, whichever it is at a particular locality tends to exhibit a higher proportion of introgressed alleles. This pattern may be due to local ecological effects or frequency-dependent introgression.
* Oreochromis niloticus x O. aureus*	Tilapia (Cichlids)	[74]	*O. aureus*	Unidirectional introgression could also result from such demographic disequilibrium, where female parents are from the rare species. Fixation of the west mtDNA lineage into the western populations of *O. niloticus* could be due to fixation by genetic drif bottleneck, selection favouring West lineage, interactions between nuclear and mitochondrial genes, long-term unidirectional hybridization, sex-ratio of hybrid progenies, or combinations of these factors.
**Insects**				
* Heliconius erato x H. himera*	Butterfly	[75]	Both (but…)	Directionality of the crosses? The barrier to gene flow is most probably a result of divergence in mate preferences, warning colour and ecology without hybrid inviability or sterility.
* Gryllus firmus x G. pennsylvanicus*	Criquets	[76]	G. pennsylvanicus	Prezygotic barriers (the hybrids from the reciprocal cross are non viable) but authors brang up (but could not test) a more general scenario where continuous input of females of one species into populations of a second species can also lead to apparent differential introgression.
* Drosophila differens x D.planitibia*	Fruit flies	[77]	*D. planitibia*	Phylogeny is partially consistent with the courtship behavioral hypothesis which states that females from derived populations will mate preferentially with males from ancestral populations, while the converse is not true. Such founder events imply the establishment of new populations by small numbers of flies-in the extreme case, perhaps a single gravid female.
**Mammals**				
* Mus musculus x M. domesticus*	Mouse	[78]	*M. domesticus*	A single *M. domesticus* individual hybridizing with *M. musculus* could be the cause of the replacement of musculus mtDNA by domesticus mtDNA in this population.
* Lepus timidus x L. europeus*	*Hare*	[36]	*L. timidus*	The females of derived species might be less discriminating than those of ancestral species (referenced in Wirtz 1999)
		[37]	*L. europeus*	Our observation differs from previous observations of mtDNA introgression in Sweden and Iberia, and provides further support for a reticulated mode of introgression within the genus Lepus.
* Tamias ruficodus x T. amoenus*	Chipmunks	[79]	*T. ruficodus*	Sexual isolation. Convergence or incomplete sorting of ancestral polymorphisms cannot be dismissed with these data.

Modified from Wirtz [21] and Avise [26]. List of some hybrid species, the mitochondrial DNA of which has been compared with that of the two parental species. This table includes only studies in which at least five hybrids were genotyped (the probability of sampling five individuals with the same type of mtDNA in a population with equal proportions of the two types of mtDNA is 0.064, two-tailed binomial test).

### Hybridization and life history traits (figures 7 and 8)

**Figure 7:**
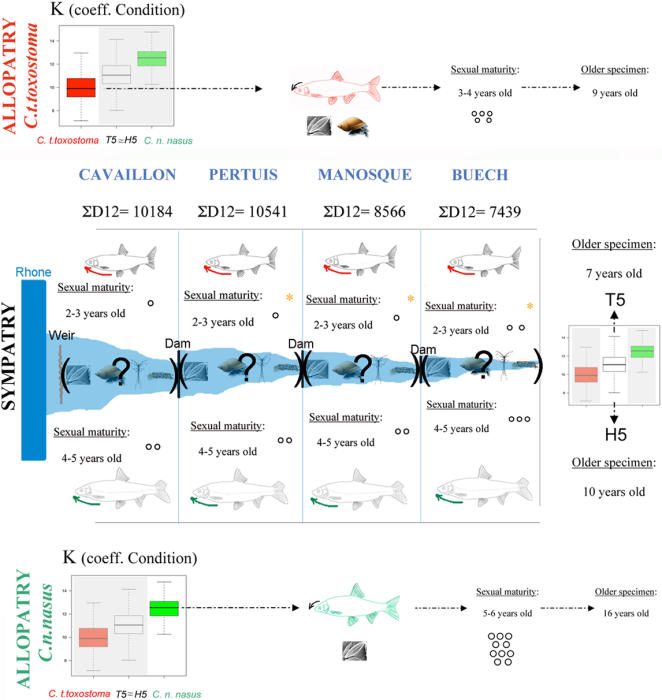
LIFE HISTORY TRAITS EVOLUTION THROUGH THE HYBRID ZONE. Global summary of changes in ecological and morphological traits in the hybrid zone with respect to the reference populations (see the text for more detail). Arrows indicate the overall trend for overall changes in snout orientation, “?” indicates that we could not strictly determine the diet of the *Chondrostoma* specimens in the hybrid zone, * indicates a significant difference, the other tests could not be done because the sample was too small, but we extrapolated a trend. “ΣD12” indicates the sum of degree-days above 12°C for each station (mean established for an 8-year data set) Chappaz (personal communication).

**Figure 8:**
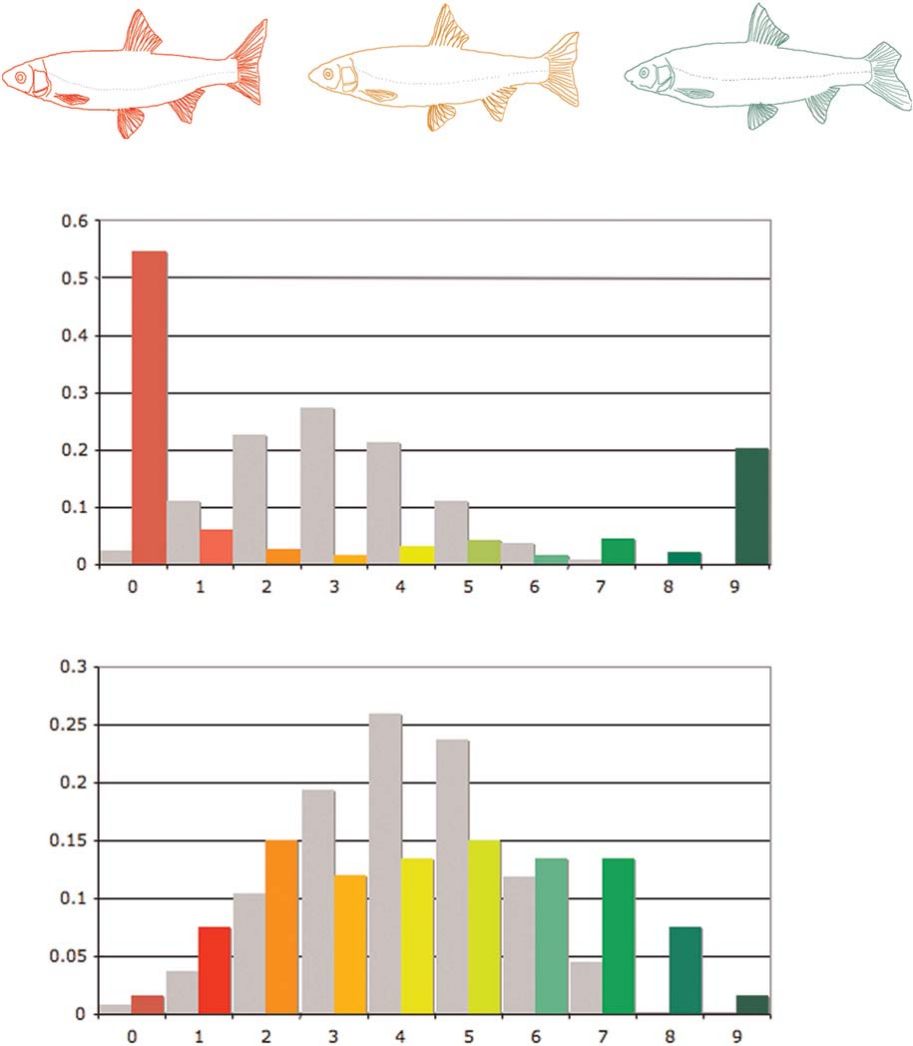
MORPHOLOGY AND GENOME DILUTION . The graphs show the distribution of individuals for each genome dilution class (x-axis) as a function of i) their probability based on numbers of specimens; y-axis (first graph) ii) their probability based on numbers of combinations; y-axis (second graph). The color gradient (observed values) extends from red (T5) to green (H5). The intermediate morphologies and genomic combinations are shown in yellow-orange, with theoretical values shown in gray (see the text for more detail). The fish morphologies shown are a simplistic representation of the morphological gradient (not all intermediate morphologies are represented).

We observed a significant change in the size of individuals, with, in particular, T5 specimens being smaller than the *C. t. toxostoma* specimens sampled from reference populations. This change in size may have limited competition with the invasive *C. n. nasus* species. However, the coefficients of condition (species effect in the allopatric zones but no genomic combination effect in the hybrid zone) obtained could be interpreted as showing convergence in the feeding of the two species in the Durance or homogeneous food assimilation. No such convergence was assumed in published data obtained for allopatric zones [38, 39 for *C. t. toxostoma* in the Rhone and Garonne Basins, respectively; 40 for *C. n. nasus* in the Rhone Basin]. Moreover, our hypotheses seem to be confirmed by the first results obtained in stable isotope (^13^C and ^15^N) studies on 168 Durance *Chondrostoma* specimens for which we can identify no species, station, or year effect.

T5 individuals reached sexual maturity at a significantly younger age than reference *C. t. toxostoma* individuals (2–3 years old, rather than 3–4 years old). A similar trend was observed for H5 and *C. n. nasus* (4–5 years old rather than 5–6 years old), but the sample size was too small for confirmation. Hybrid zone females also had significantly fewer eggs than reference population females (figure 7), consistent with previous findings [41], [42]. Changes in temperature may be responsible for changes in age at sexual maturity and the number of eggs per female [as in 43]. Thus, interspecies competition is not the only factor responsible for ecological displacement; environmental pressure also plays a key role in the evolution of sympatric species.

We observed a morphological gradient, passing from an upper limit of about 80% of alleles from one species, through an intermediate morphology to the morphology of the other species (figure 8). However, we identified changes in the general body shape of hybrid zone individuals, with a shift in mean morphology for all genomic combinations of the hybrid zone, regardless of sampling station or the age or sex of specimens. Surprisingly, this shift was towards the *nasus* morphology rather than that of *toxostoma*, which is presumably better adapted to its niche. This shift in morphology may be associated with the Durance River rather than a direct effect of hybridization, because the level of hybridization differed between stations, making it highly unlikely that the direction of change would be the same at all stations. This result tends to confirm the influence of environment on the *Chondrostoma* complex, as suggested by Peacor *et al*. [44]. So, is the regular flow of water from upstream to downstream (via the dams) a major factor in the global unidirectional deformation of body shape regardless of genomic combination, and of the size and age of specimens? Studies of other cyprinid hybrid zones would be required to test these hypotheses and to evaluate the role of ecological conditions in speciation [45].

### Crossroads between population genomic and ecological approaches

Our results indicate that the interspecies population dynamics of hybrid zones are governed by a genomic component and an ecological component, which may be difficult to dissociate. It is widely thought that shoal members benefit most from being in phenotypically uniform shoals [20]. Could this account for the unidirectional effect of the Durance River on the morphology of the two *Chondrostoma* species?

A previous study [46] demonstrated that shoaling behavior has a genetic component and provided a preliminary estimate of its heritability in zebrafish (*Danio rerio*). More recently [47], QTL for shoaling and boldness have been identified on chromosome 16 of *Danio rerio*. This is particularly interesting because the *Tpi 1b* gene is also present in this linkage group. This result supports our biological conclusions and confirms the need to focus on these aspects for future “eco-geno” studies of hybrid zones.

## MATERIALS AND METHODS

### Collection of samples and area studied

**Figure 9:**
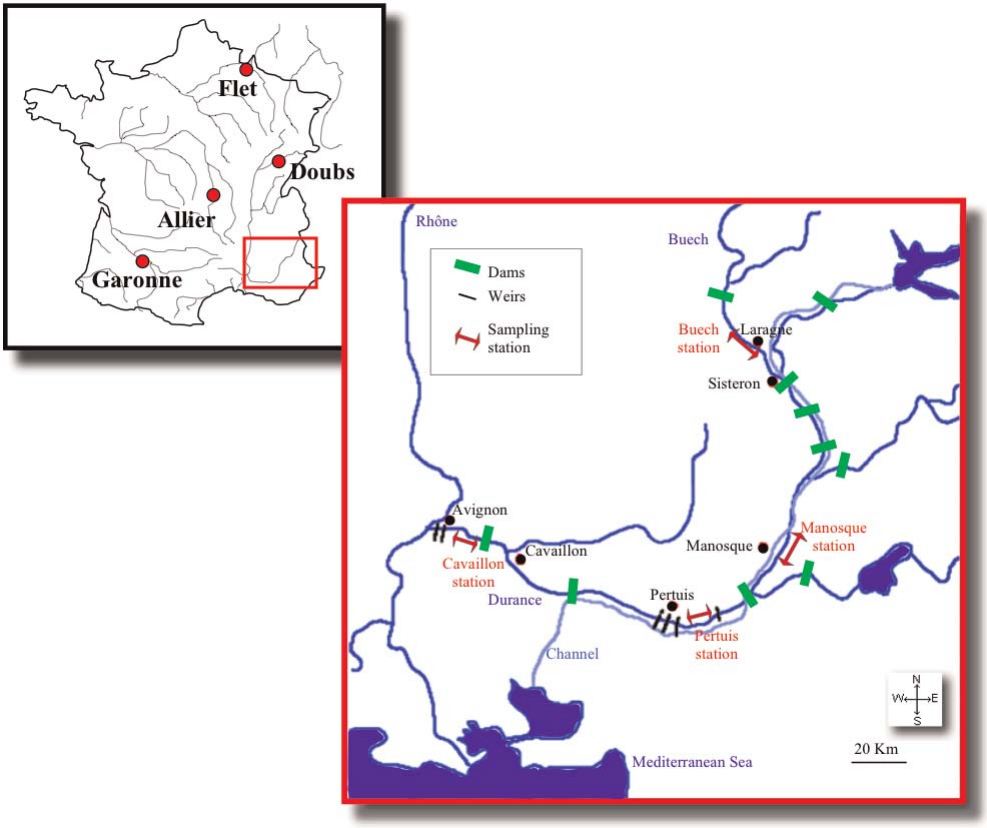
GEOGRAPHICAL AREA STUDIED. Allopatric zone: Garonne (*C. t. toxostoma*); Flet (*C. n. nasus*); parapatric zones: Doubs (*C. t. toxostoma*); Allier (*C. n. nasus*) and hybrid zone: the River Durance and the four sampling stations: Buech, Manosque, Pertuis and Cavaillon.

For each of the two species, a population in allopatry (38 individuals from the Garonne for *C. t. toxostoma*; 33 individuals from the Flet for *C. n. nasus*) and a population in parapatry (30 individuals from the Doubs for *C. t. toxostoma*; 27 individuals from the Allier for *C. n. nasus*) were analyzed and treated as reference populations (i.e. “pure” representative specimens of the two species; figure 9). The choice of these reference populations was dictated by a previous molecular study [9], in which these populations displayed the largest range of variability. The River Durance (hybrid zone) is the last major affluent of the Rhone on the eastern side. This river has many dams and weirs isolating different parts of the river and preventing the migration of freshwater fauna. Construction of the major dams began around 1950, after *C. n. nasus* began to colonize the Durance.

In the hybrid zone, 642 individuals were sampled in 2001 and 853 in 2002, at four independent stations (figure 9). Each station was sampled repeatedly between March and the end of June (Appendix S1). An identifier was attributed to each fish, in order of sampling.

### Molecular marker

Total genomic DNA was extracted from muscle tissue or from a piece of caudal fin, using a standard phenol chloroform protocol [48].

#### mtDNA

A 610 bp fragment of the cytochrome *b* mtDNA sequence was amplified using primers and an amplification protocol described elsewhere [10]. The single strand was used as a template for the sequencing of 532 bp with an automated DNA sequencer (Macrogen Inc.) (Genbank accession number EF363338-EF363452).

#### Nuclear introns

We first tested 13 introns (Gilles and Costedoat unpublished data). Each was used to search the Genbank database for potential paralogs representing a major risk in our study of hybridization between species of the Cyprinidae family, due to the presence of 3R replication events in teleosts in particular [49], [50], [51]. The position of the gene was systematically checked, based on the chromosome map of *Danio rerio* (Teleostei; Cyprinidae). We finally chose to study four introns (ribosomal protein gene S7 intron 1; triose phosphate isomerase 1b, Tpi1b intron 4; α-tropomyosin, α-Trop intron 5; and recombination activating gene 1, RAG 1 intron 2) because they presented a sufficiently high level of polymorphism and alleles capable of discriminating between the two *Chondrostoma* species. Sequence variation within and between populations and species was characterized by analyzing single-stranded conformational polymorphisms (SSCP) [52], [53], [54], using [α^32^P]-dATP (IRSN Corp.)-labeled DNA fragments. The amplification protocols and primers are presented in the Dataset S3. As the SSCP method requires the migration of relatively small fragments, the sequence lengths of the intron amplicons were 392 bp for RAG-1 (Genbank accession number EF363453-EF363458), 186 bp for S7 (Genbank accession number EF363459-EF363466), 327 bp for Tpi1b (Genbank accession number EF363467-EF363472) and 224 bp for α-Trop (Genbank accession number EF363473-EF363479).

Mitochondrial and nuclear allele affiliations and phylogenetic reconstructions are presented in the Dataset S4.

### Specimen identification and Hardy-Weinberg equilibrium

Each specimen of the hybrid zone was identified by combining the alleles observed for each of the five molecular markers (*cf.* Dataset S4). In “pure” *C. n. nasus* specimens, all five markers (*cytochrome b* plus the four introns) originated from *C. n. nasus* (the reciprocal applied to “pure” *C. t. toxostoma* individuals). We could potentially identify 162 genomic combinations (the two “pure” or parental combinations plus 160 hybrid or introgressed combinations). We used a previously described approach ([55] and [56]) to investigate non randomness in genotype frequencies (departure from Hardy-Weinberg expectations and from linkage equilibrium). We used chi-square tests to check for non randomness in genotype frequencies, with Benjamini-Hochberg correction for multiple comparisons. Indeed, as we carried out as many tests as there were combinations for the Hardy-Weinberg test of equilibrium, (i.e. 162), we had to control for false positives. We used the false discovery rate (FDR) and its majoration proposed by Benjamini and Hochberg [57]. Let (Pi)_i_ be the ordered P-value for the 162 tests,
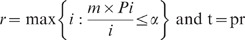
3Then we have FDR (t) ≤ α

In practical terms, each test giving a P-value Pi such that 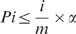 was declared significant.

With this approach, we were able to assign the different genomic combinations to three major groups: combinations that were overrepresented, combinations that were underrepresented and combinations present in the expected proportions. This approach also permitted robust use of the statistical models.

### Morphological markers

We ensured that the morphological ranges of the two species were as large as possible by also adding two other allopatric populations—32 *C. t. toxostoma* specimens from the Berre (a Mediterranean coastal river) and 24 *C. n. nasus* specimens from Romania.

Morphology was analyzed by studying four meristic characters (numbers of dorsal, anal and pelvic fins, rays and number of scales along the lateral line) and through a morphometric approach, based on the landmarks method [58]. We defined 21 homologous landmarks on the body (figure S5). All landmarks were digitized, using TpsDig software [59]. Landmark-based geometric morphometric methods were used to capture information about shape, by obtaining the x and y coordinates of homologous landmarks. Differences in the sets of coordinates between specimens due to scaling, rotation and translation were eliminated by a typical geometric morphometric approach [60], [61] in which the specimens were placed in a procruste superimposition on the iteratively estimated mean reference form, using the generalized procruste analysis (GPA) procedure. Points representing landmark configurations were then projected into Euclidean tangent space approximating the curved shape space.

Morphological variables (both meristic and plastic variables) were first analyzed for the allopatric and parapatric populations, and then for the hybrid zone. For each analysis, a MANCOVA was performed on size, age, sex, station and species (or genetic class) effects. Size was calculated as centroid size (i.e. the squared root of the sum of squared distances between each landmark and the centroid (centre of gravity) of a landmark configuration). For significant effects, discriminant analysis was then performed, with deformation grids used for plastic variables, using Bookstein's [62] thin plate spline algorithm. All statistical analyses were performed using R [63]; deformation grids were visualized with the tpsgrid function of the R shape package [60].

### Life history traits

Weight, size, age and sex were determined for each specimen.

The relative robustness, or degree of well-being, can be expressed as the “coefficient of condition” [64]. Variations in the coefficient of condition primarily reflect the state of sexual maturity and nourishment. The formula most often used is: K = W/L^3^ (W = the weight in grams; L = the standard length in decimeters). We carried out an analysis of variance to determine whether the mean coefficient of condition differed significantly i) between the two *Chondrostoma* species ii) between the different populations of each of the *Chondrostoma* species (allopatry and parapatry) and iii) between the reference populations and the hybrid zone populations.

As both species are protected, the individuals sampled in 2002 were analyzed directly in the field and then released. However, the *Chondrostoma* species do not display sexual dimorphism and have no sexual chromosome. Sex was therefore determined by the canulation method for this sampling year (Stolzenberg personal communication). As a result, it was very difficult to determine the sex of the animals in some sampling campaigns and for some specimens.

### Variable interactions

We analyzed interactions between the various qualitative variables, using chi-square tests or log-linear models [10]. For quantitative variables (coefficient of condition and morphology), interactions were analyzed by variance-based methods—ANOVA and MANCOVA, respectively.

## Supporting Information

Appendix S1 SAMPLING INFORMATION. Sampling campaigns and sample size for each Durance station.
(0.16 MB DOC)

Dataset S1 Molecular marker history and allele distribution.
(0.06 MB DOC)

Dataset S2 Hybrid zone and life history traits
(0.04 MB DOC)

Dataset S3 Nuclear amplification protocols and SSCP method
(0.04 MB DOC)

Dataset S4 Genetic analysis
(0.05 MB DOC)

Figure S1 MITOCHONDRIAL C.T. TOXOSTOMA NETWORK. C.t. toxostoma mtDNA haplotype network.
(2.44 MB TIF)

Figure S2 MITOCHONDRIAL C.N. NASUS NETWORK. C.n. nasus mtDNA haplotype network.
(1.12 MB TIF)

Figure S3 PHYLOGENETIC RECONSTRUCTION. Neighbor-Joining (with Kimura-2-parameter distance model) based on the sequences obtained for each of the four introns. All the topologies were tested by non-parametric bootstrap resampling (500 replicates).
(0.86 MB TIF)

Figure S4 EVOLUTIONARY HISTORY OF THE NUCLEAR INTRONS. Left side: summary of the phylogentic relationships between haplotypes of the two species (C. nasus and C. toxostoma) obtained with NJ and parsimony methods. Right side: haplotype relationships under haplotype network representation. C. toxostoma haplotypes are in red, C. nasus haplotypes are in green. For network reconstruction, the relationships between the two species haplotypes required a decrease in the parsimony limit (90%) obtained with TCS software. The arrow corresponds to the root as observed on the NJ trees. For the S7 intron, the Rh2 haplotype corresponds to a Romanian C. n. nasus haplotype.
(1.31 MB TIF)

Figure S5 LANDMARKS. The 21 chosen landmarks used for morphometric analysis.
(0.51 MB TIF)

Figure S6 DISCRIMINANT ANALYSIS REALIZED ON PLASTIC MORPHOLOGICAL CHARACTERS. Illustration of the Durance tendency. T = C.t.toxostoma in allopatry/paraptry; H = C.n.nasus in allopatry/paraptry; H5 = C.n.nasus in the hybrid zone; T5 = C.t.toxostoma in the hybrid zone and the different hybrid groups. The DA was carried out with all the groups, but we illustrate only the variance of the T (in blue), T5 (in red), H (in green) and H5 (purple) groups.
(1.19 MB TIF)

Figure S7 VARIANCE OF THE COEFFICIENT OF CONDITION. Graphic representation of variance analyses of the coefficient of condition in sympatry. A) by year; B) as a function of sex F = female; M = male; I = immature, C) by station, 17 = Buech; 18 = Manosque; 19 = Pertuis; 20 = Cavaillon, D) by age and E) by genomic combination, H5; H4T; Hi4; T4i; T5; -1 = under-represented combinations; 0 = combinations in the expected proportions; 99 = over-represented combinations; *significant value.
(1.59 MB TIF)

Table S1 MTDNA HAPLOTYPE DISTRIBUTION. Letter A = C. toxostoma; B = C. nasus and y = hybrid zone haplotypes. Haplotype diversity (H) and nucleotide diversity (Pi) were calculated for each population.
(0.09 MB DOC)

Table S2 NUCLEAR ALLELES DISTRIBUTION. Genotype distribution of the 4 introns (S7; α-Trop; Rag; Tpi) as a function of the population sampled (reference populations and hybrid zone populations).
(0.14 MB DOC)

Table S3 CROSS VALIDATION PROCEDURE FOR MORPHOLOGICAL CHARACTERS. C.n.n = C.n.nasus in reference zones; C.t.t = C.t. toxosotoma in reference zones. -1 = underrepresented combinations; 0 = combinations in expected proportion; 1 = overrepresented combinations; H5 = C.n.nasus from hybrid zone; T5 = C.t. toxosotoma from hybrid zone; H4T; Hi4; T4i = hybrid combinations (cf text for more explanations).
(0.11 MB DOC)

Table S4 MANCOVA ON MORPHOLOGICAL DATA SET. Order-3 analysis. Cond: Coefficient of condition, Gen. Group: Genomic group (cf. text for more details).
(0.13 MB DOC)

Table S5 ANOVA ON COEFFICIENT OF CONDITION DATA SET.
(0.03 MB DOC)

Table S6 AGE DISTRIBUTION. Distribution of hybrid zone combinations as a function of genome dilution and age (data are in percentages).
(0.06 MB DOC)

Table S7 SEX RATIO IN THE HYBRID ZONE. References did not indicate a sex ratio significantly different from 1 for the two species studied in allopatric populations [1], [2]. F = female, M = male and I = indeterminate. 1) Gozlan RE (1998) Environmental biology and morphodynamics of the sofie Chondrostoma toxostoma (Cyprinidae), with emphasis on early development. PhD, Université Toulouse, France. 196p. 2) Nelva-Pasqual A (1985) Biogéographie, démographie et écologie de Chondrostoma nasus nasus (L., 1758) Hotu (Poisson, Téléostéen, Cyprinidé). PhD Université Claude-Bernard-Lyon I. 340p.
(1.51 MB DOC)

Table S8 VARIABLES INTERACTIONS. A) order-two interactions (chi2); B) order-three interactions (log-linear model) and main associated results.
(0.12 MB DOC)
