# Supplementary material for: Novelties in Hybrid Zones: Crossroads between Population Genomic and Ecological Approaches
Source: PLoS One. 2007 Apr 4;2(4):e357. doi: 10.1371/journal.pone.0000357 (PMC1831490; doi:10.1371/journal.pone.0000357)
Supplement: Appendix S1 — SAMPLING INFORMATION. Sampling campaigns and sample size for each Durance station. (0.16 MB DOC) [file pone.0000357.s001.doc]

Appendix S1:
