# Supplementary material for: Novelties in Hybrid Zones: Crossroads between Population Genomic and Ecological Approaches
Source: PLoS One. 2007 Apr 4;2(4):e357. doi: 10.1371/journal.pone.0000357 (PMC1831490; doi:10.1371/journal.pone.0000357)
Supplement: Dataset S1 — Molecular marker history and allele distribution. (0.06 MB DOC) [file pone.0000357.s002.doc]

**Dataset S1-Molecular marker history and allele distribution.**

**mtDNA.** Based on the alignment and analyses of 532 bp of the *cytochrome b* gene and considering the 1623 specimens (allopatric, parapatric and hybrid zone specimens), we found 85 *C. t. toxostoma* haplotypes and 31 *C. n. nasus* haplotypes (Table S1). Only six of the 85 *C. t. toxostoma* haplotypes and two of the 31 *C. n. nasus* haplotypes were found in the respective reference populations and the hybrid zone (Table S1). In the two cases for *C. n. nasus* haplotypes, the shared haplotypes corresponded to the main haplotypes of the hybrid zone. However, haplotype and nucleotide diversities did not differ significantly between the reference populations and the hybrid zone population, except for the Flet population (Table S1). These results may be accounted for by the larger number of specimens sampled from the Durance.

The phylogenetic relations represented by haplotype networks (TCS software [1] (figures S1 and S2) showed that most of the rare haplotypes present in the hybrid zone correspond to a one-step mutation from common haplotypes. The *C. t. toxostoma* network presented numerous cycles translating the presence of alternative evolutionary pathways, particularly between specific Manosque and Pertuis haplotypes. The A4 haplotype is defined as the ancestral one (figure S1) and was the only haplotype shared by the two reference populations and all the populations of the hybrid zone. The *C. n. nasus* network was simpler, with no cycle. However, in contrast to what was found for the *C. t. toxostoma* network, the ancestral haplotype B2 (figure S2) was not shared by the two reference populations, but was also present in the Allier population and in the hybrid zone. This may be accounted for by calculation of the network "root". Indeed, calculation of the root depends less on the absolute number of populations sharing this haplotype and more on its relative proportion within the data set, its position in the network (interior or apex), and the number of haplotypes to which it is directly connected [2].

**Introns.** Alleles were affiliated to one of the parent species based on the profiles obtained for the reference populations. When an allele was found only in the hybrid zone, its identity could be established reliably only after sequence analysis and phylogenetic reconstruction. The affiliation of the allele D of the S7 intron was the only real problem (figure S3, table S2). The topologies obtained with the various phylogenetic methods were unclear (figure S3 only the NJ with the Kimura 2-parameter distance model is presented). We chose the most parsimonious biological identification, comparing, for the individuals presenting this allele, the results obtained for the other markers and the sampling station. Allele D of S7 was thus regarded as affiliated to *C. n. nasus*. The use of outgroup cyprinid species for each of our introns made it possible to check that all the hybrid individuals were hybrids between the two *Chondrostoma* species only.

The genotypic distribution of introns by population is presented in table S2. Diversity was greatest for the S7 intron, with 30 different genotypes, whereas we found 17, 13 and 11 genotypes, respectively, for the α-Trop, RAG-1 and Tpi1b introns. Unlike the other introns, S7 presented more *C. n. nasus* alleles than *C. t. toxostoma* alleles and more different hybrid genotypes (i.e. heterozygous genotypes presenting a *C. n. nasus* allele and a *C. t. toxostoma* allele).

The different topologies obtained with the four introns perfectly illustrate the differences between the history of species and the history of genes (figure S3). From a phylogenetic point of view, Tpi 1b appeared to be the only interesting marker, because the speciation event coincided with the dichotomy of the *C. t. toxostoma* and *C. n. nasus* alleles (figure S3). This conclusion is based on the properties of nuclear markers, which may evolve only one tenth as fast as mitochondrial markers [3], and on the length of the sequences that can show heterogeneity in the rate of substitution when too short. Figure S4 summarizes, under tree reconstruction and network representations, the phylogenetic evolution of the various alleles from each intron.

**References**

1. Clement M, Posada D, Crandall KA (2000) TCS: a computer program to estimate gene genealogies. Mol Ecol 9: 1657-1659.
2. Castelloe J, Templeton A (1994) Root probabilities for intraspecific gene trees under neutral coalescent theory. Mol Phyl Evol 3: 102-113.
3. Wilson AC, Cann RL, Carr SM, *et al*. (1985) Mitochondrial-DNA and 2 perspectives on evolutionary genetics. Biol J Linn Soc 26: 375-400.
