# Supplementary material for: Novelties in Hybrid Zones: Crossroads between Population Genomic and Ecological Approaches
Source: PLoS One. 2007 Apr 4;2(4):e357. doi: 10.1371/journal.pone.0000357 (PMC1831490; doi:10.1371/journal.pone.0000357)
Supplement: Dataset S2 — Hybrid zone and life history traits (0.04 MB DOC) [file pone.0000357.s003.doc]

**Dataset S2-Hybrid zone and life history traits**

**Morphology**. Result precisions: We first assessed whether morphological variables (meristic and plastic variables) could be used for species and/or population identification. We tested species and population nested effects on the morphological variables for the reference populations (allopatric and parapatric populations, cf. material and methods). Both these effects were significant. For meristic characters: i) species effect: vector coefficient of correlation η^2^=0.890; pseudoF=715.22; df=4.35; *p* <10^-15^ and ii) population effect: vector coefficient of correlation η^2^=0.938; pseudoF=31.97; df=44.13; *p* <10^-15^. For plastic characters: i) species effect: vector coefficient of correlation η^2^=0.930; pseudoF=32.9; df=38; *p* <10^-15^ and ii) population effect: vector coefficient of correlation η^2^=0.9992; pseudoF=11.89; df=152; *p* <10^-15^.

**Coefficient of condition**. Remark: most of the individuals in allopatric populations were less than three years old. We therefore analyzed coefficient of condition only for the 1-, 2- and 3-year age classes, to allow comparison with reference populations. Furthermore, we pooled the H4T (14 individuals in this age range) and Hi4 (10 individuals) genomic classes with the 99 class (all the other over-represented combinations), due to small sample sizes.

Interactions of age and genomic class on coefficient of condition main results: Age (F = 6.92; df=2.905 *p*=0.0158) and genomic class (F = 15.95; df=7.905; *p* =0023) had significant effects on coefficient of condition. The three effects (age, station, and interaction age x station) were significant: coefficient of condition decreased with increasing age for *C. n. nasus*, *C. t. toxostoma* and H5, whereas it increased with age for all other classes. Paired comparisons (with Benjamini-Hochberg correction) were only significant if they included an allopatric population.
