# Supplementary material for: Novelties in Hybrid Zones: Crossroads between Population Genomic and Ecological Approaches
Source: PLoS One. 2007 Apr 4;2(4):e357. doi: 10.1371/journal.pone.0000357 (PMC1831490; doi:10.1371/journal.pone.0000357)
Supplement: Dataset S3 — Nuclear amplification protocols and SSCP method (0.04 MB DOC) [file pone.0000357.s004.doc]

**Dataset S3-Nuclear amplification protocols and SSCP method:**

Polymerase chain reaction (PCR) conditions for S7, Tpi1b, and RAG-1: 94°C, 1 min; 55°C, 1 min; 72°C, 2 min; 30 cycles. For α-Trop, the amplification conditions were 94°C, 1 min; 50°C, 1 min; 72°C, 2 min; 30 cycles. The primers used for intron amplification were, for S7 (intron 1): S7R4F 5'-TAACGGCATGCTAAGAACAC-3' (forward) and S7R4R 5'-TACAATCTGATGCGTCTG- 3' (reverse) (Gilles and Costedoat unpublished data); for Tpi1b (intron 4): TpiF2 5'-GCATYGGGGAGAAGCTRGA-3' (forward) and TpiR3 5'-TCCCGGAGCTTGTCATGCAC-3' (reverse) (Dowling and Gilles personal communication); for RAG-1 (intron 2): RIF2 5'- AGCCCTTGTATCTGCAAATG-3' (forward) and RIR2 5'- ACCGTGCGGTACATCTTGTG-3' (reverse) (Gilles and Costedoat unpublished data); and for α-Trop (intron 5) TROPF 5'- GAGTTGGATCGCGCTCAGGAGCG-3' (forward) and TROPR 5'- CGGTCAGCCTCCTCAGCAATGTGCTT-3' (reverse) [1].

SSCP gels (8% acrylamide) were run at room temperature for 18 h at 500 volts. Ambiguous haplotypes or haplotypes difficult to score across the gel were re-run on additional gels (or sequenced) until all haplotypes were resolved.

At least two representatives of each SSCP variant were sequenced, using an automated DNA sequencer (Macrogen Inc.). Amplification parameters for generating PCR products for sequencing were as described above, except that [α^32^P]-dATP was not used and reaction volumes were larger (25 μl instead of 5 μl for SSCP).

**Reference**

1. Friesen VL, Congdon BC, Kidd MG, Birt TP (1999) Polymerase chain reaction (PCR) primers for the amplification of five nuclear introns in vertebrates. Mol Ecol 8: 2147-2149.
