# Supplementary material for: Novelties in Hybrid Zones: Crossroads between Population Genomic and Ecological Approaches
Source: PLoS One. 2007 Apr 4;2(4):e357. doi: 10.1371/journal.pone.0000357 (PMC1831490; doi:10.1371/journal.pone.0000357)
Supplement: Dataset S4 — Genetic analysis (0.05 MB DOC) [file pone.0000357.s005.doc]

**Dataset S4-Genetic analysis**

**Linkage disequilibrium test (for the four introns)**. The four genes studied are on four different chromosomes (LG25 for RAG-1, LG20 for S7, LG16 for Tpi1b, LG7 for α-Trop), and may therefore be considered independent. However, we checked the independence of the introns by means of the linkage disequilibrium test, as implemented in GENETIX software [1], for the reference populations (allopatric and parapatric populations) and then for the hybrid zone populations. In both reference and hybrid zone populations, the introns were not in linkage disequilibrium and were therefore considered independent.

**Allele affiliation**. The affiliation of the different alleles present in the hybrid zone to the *C. t. toxostoma* or *C. n. nasus* species was determined by comparing the migration profile of the specimens from allopatric and parapatric populations. If the allele was not present in the reference populations, only genetic analyses (phylogenetic reconstruction) could be used to determine affiliation.

**Phylogenetic reconstructions**. We carried out pairwise sequence comparisons, using the neighbor-joining method, based on the Juke & Cantor and Kimura-2-parameter distance model (Mega2 software [2]), on *cytochrome b* and the intron data set. We tested the topology of the tree produced, by a non-parametric bootstrap method. We also used the maximum parsimony reconstruction method (as implemented in Paup*4.0 version beta [3]), involving heuristic search, 10 random additional sequences and TBR branch swapping. Estimates of the robustness of each clade were obtained using 1000 non-parametric bootstraps.

**Nested clade analysis (NCA)**. We performed NCA, as described by Templeton *et al.* [4] for the mitochondrial data set and for each of the four introns. Networks were constructed based on a statistical parsimony procedure, as implemented in TCS 1.18 [5].

References

1. Belkhir K, Borsa P, Chikhi L, Raufaste N, Bonhomme F (1996-2002) GENETIX 4.04, logiciel sous windows TM pour la génétique des populations. In: Laboratoire Génome, Populations, Interactions.
2. Kumar S, Tamura K, Jakobsen IB, Nei M (2001) MEGA2: molecular evolutionary genetics analysis software. Bioinformatics 17: 1244-1245.
3. Swofford, DL (2003) PAUP*: Phylogenetic analysis using parsimony (* and other methods), version 4.0b 10. Sinauer Associates, Sunderland, Massachusetts.
4. Templeton AR, Routman E, Phillips CA (1995) Separating population structure from population history: a cladistic analysis of the geographical distribution of mitochondrial DNA haplotypes in the tiger salamander, *Ambystoma tigrinum*. Genetics 140: 767.
5. Clement M, Posada D, Crandall KA (2000) TCS: a computer program to estimate gene genealogies. Mol Ecol 9: 1657-1659.
