# Supplementary figures and images for: Novelties in Hybrid Zones: Crossroads between Population Genomic and Ecological Approaches
Source: PLoS One. 2007 Apr 4;2(4):e357. doi: 10.1371/journal.pone.0000357 (PMC1831490; doi:10.1371/journal.pone.0000357)

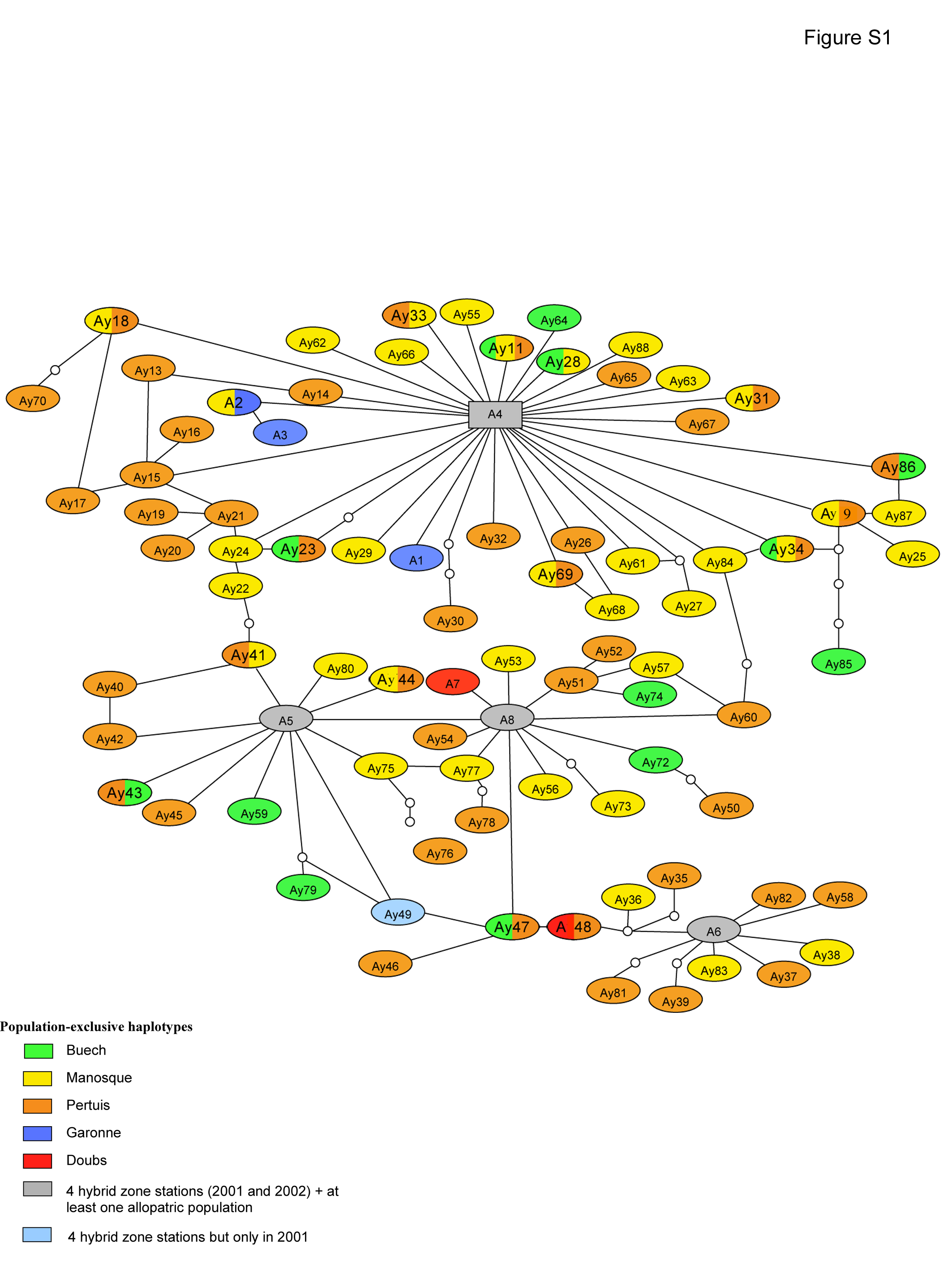

Supplement: Figure S1 — MITOCHONDRIAL C.T. TOXOSTOMA NETWORK. C.t. toxostoma mtDNA haplotype network. (2.44 MB TIF) [file pone.0000357.s006.tif]

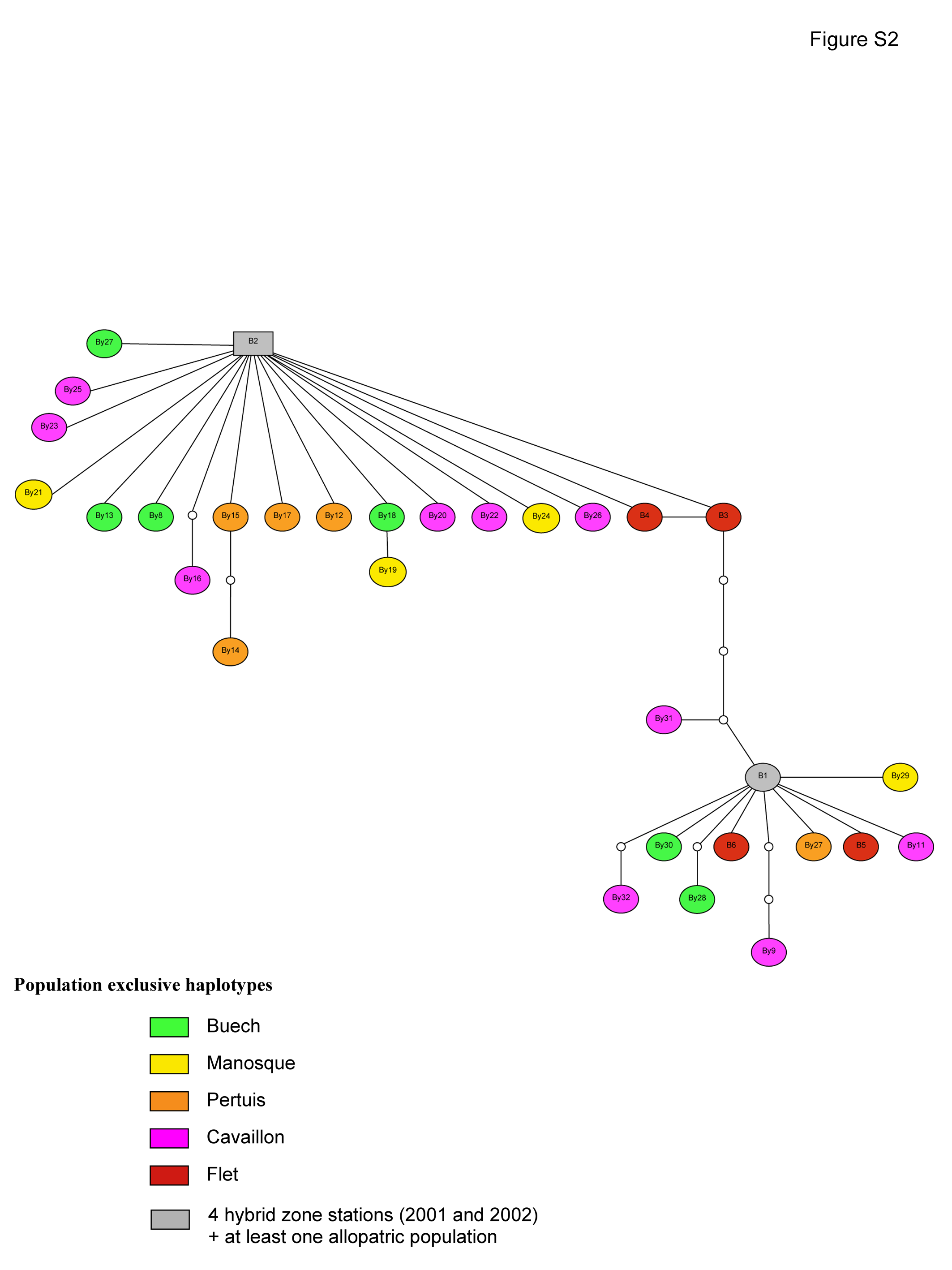

Supplement: Figure S2 — MITOCHONDRIAL C.N. NASUS NETWORK. C.n. nasus mtDNA haplotype network. (1.12 MB TIF) [file pone.0000357.s007.tif]

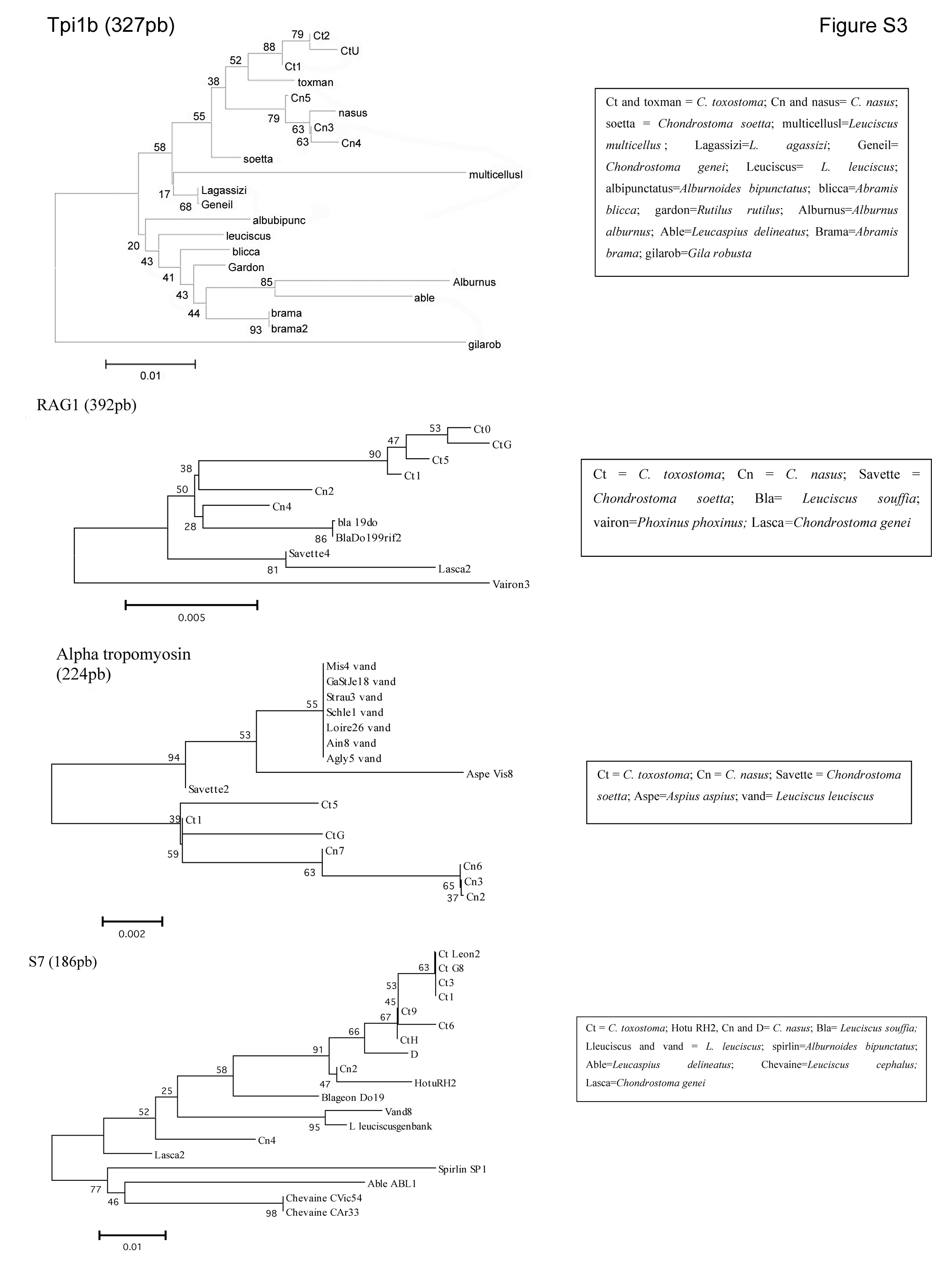

Supplement: Figure S3 — PHYLOGENETIC RECONSTRUCTION. Neighbor-Joining (with Kimura-2-parameter distance model) based on the sequences obtained for each of the four introns. All the topologies were tested by non-parametric bootstrap resampling (500 replicates). (0.86 MB TIF) [file pone.0000357.s008.tif]

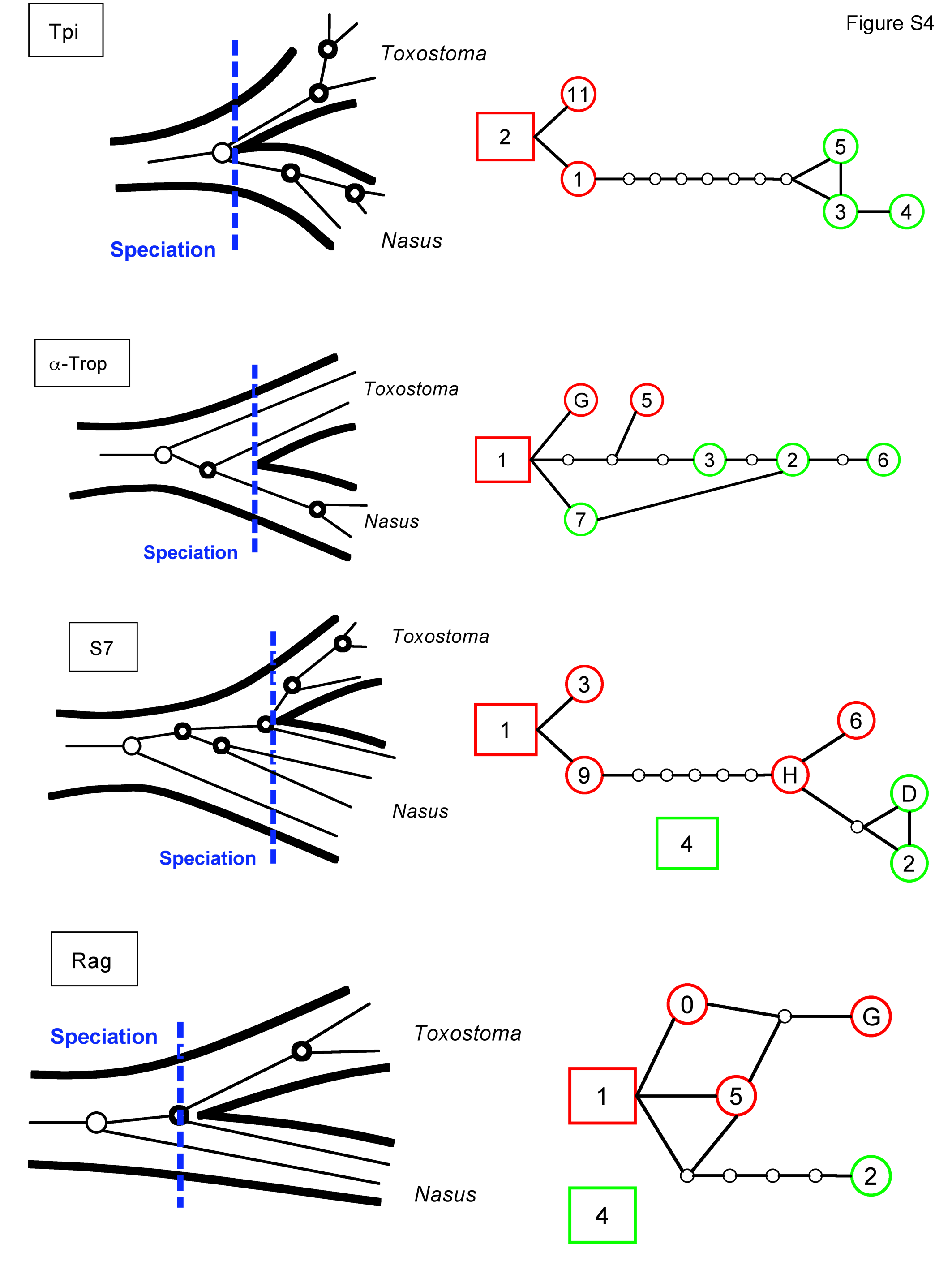

Supplement: Figure S4 — EVOLUTIONARY HISTORY OF THE NUCLEAR INTRONS. Left side: summary of the phylogentic relationships between haplotypes of the two species (C. nasus and C. toxostoma) obtained with NJ and parsimony methods. Right side: haplotype relationships under haplotype network representation. C. toxostoma haplotypes are in red, C. nasus haplotypes are in green. For network reconstruction, the relationships between the two species haplotypes required a decrease in the parsimony limit (90%) obtained with TCS software. The arrow corresponds to the root as observed on the NJ trees. For the S7 intron, the Rh2 haplotype corresponds to a Romanian C. n. nasus haplotype. (1.31 MB TIF) [file pone.0000357.s009.tif]

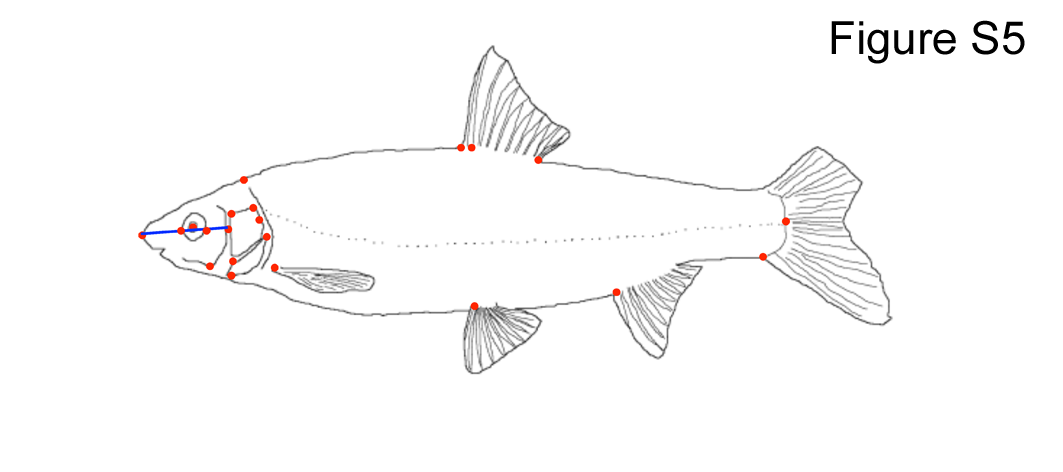

Supplement: Figure S5 — LANDMARKS. The 21 chosen landmarks used for morphometric analysis. (0.51 MB TIF) [file pone.0000357.s010.tif]

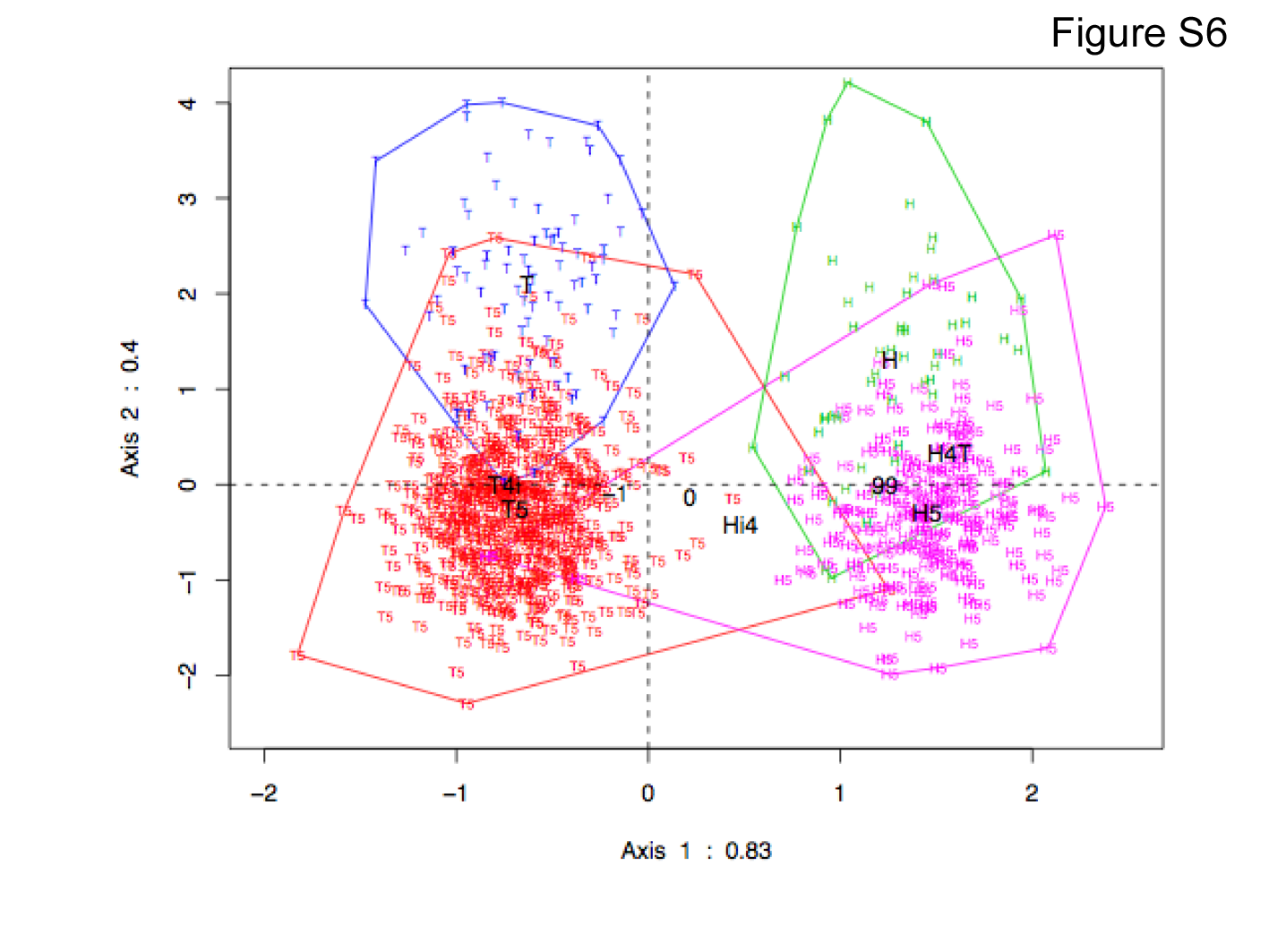

Supplement: Figure S6 — DISCRIMINANT ANALYSIS REALIZED ON PLASTIC MORPHOLOGICAL CHARACTERS. Illustration of the Durance tendency. T = C.t.toxostoma in allopatry/paraptry; H = C.n.nasus in allopatry/paraptry; H5 = C.n.nasus in the hybrid zone; T5 = C.t.toxostoma in the hybrid zone and the different hybrid groups. The DA was carried out with all the groups, but we illustrate only the variance of the T (in blue), T5 (in red), H (in green) and H5 (purple) groups. (1.19 MB TIF) [file pone.0000357.s011.tif]

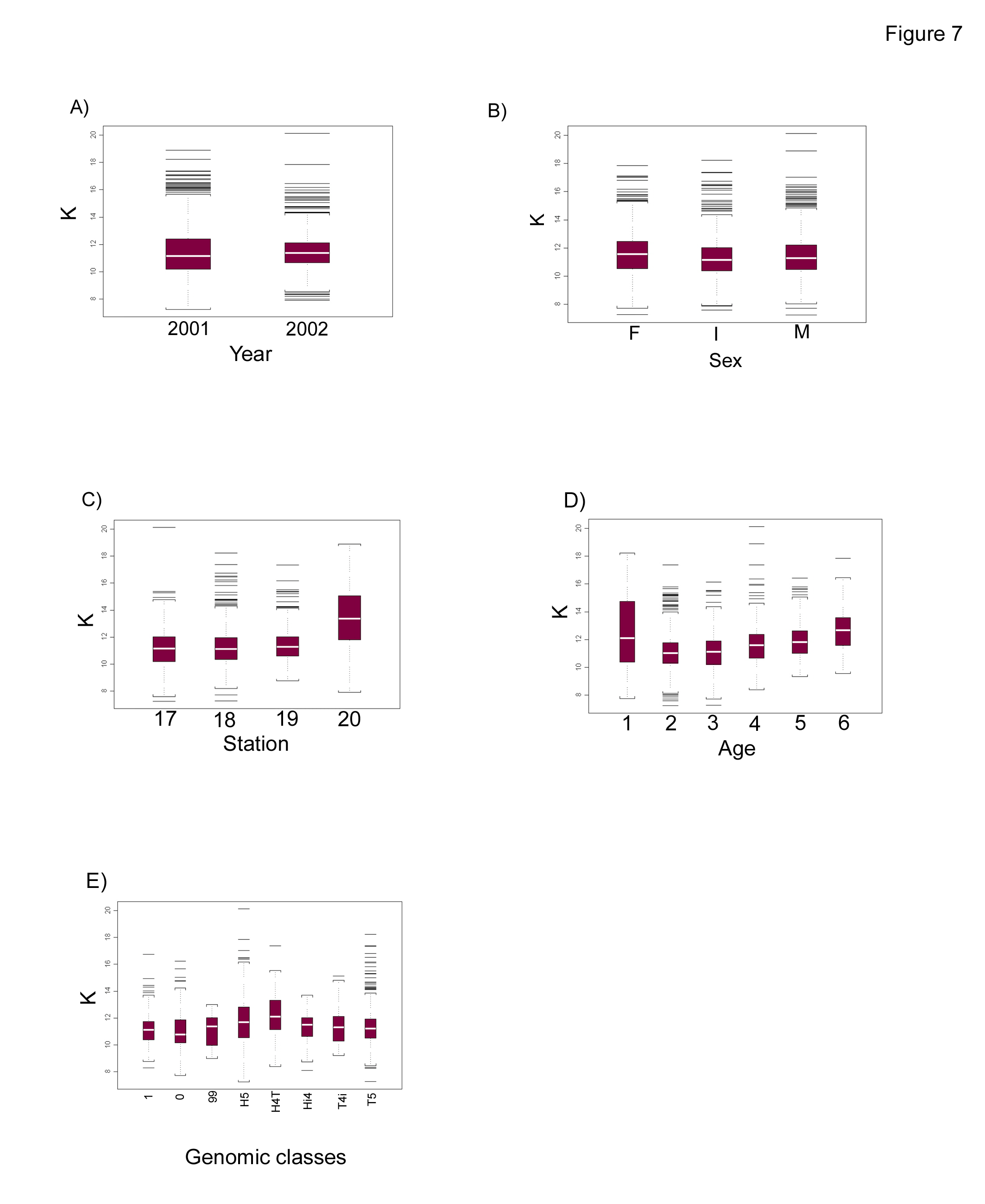

Supplement: Figure S7 — VARIANCE OF THE COEFFICIENT OF CONDITION. Graphic representation of variance analyses of the coefficient of condition in sympatry. A) by year; B) as a function of sex F = female; M = male; I = immature, C) by station, 17 = Buech; 18 = Manosque; 19 = Pertuis; 20 = Cavaillon, D) by age and E) by genomic combination, H5; H4T; Hi4; T4i; T5; -1 = under-represented combinations; 0 = combinations in the expected proportions; 99 = over-represented combinations; *significant value. (1.59 MB TIF) [file pone.0000357.s012.tif]
