# Supplementary material for: Novelties in Hybrid Zones: Crossroads between Population Genomic and Ecological Approaches
Source: PLoS One. 2007 Apr 4;2(4):e357. doi: 10.1371/journal.pone.0000357 (PMC1831490; doi:10.1371/journal.pone.0000357)
Supplement: Table S1 — MTDNA HAPLOTYPE DISTRIBUTION. Letter A = C. toxostoma; B = C. nasus and y = hybrid zone haplotypes. Haplotype diversity (H) and nucleotide diversity (Pi) were calculated for each population. (0.09 MB DOC) [file pone.0000357.s013.doc]

Table S1:
