# Supplementary material for: Novelties in Hybrid Zones: Crossroads between Population Genomic and Ecological Approaches
Source: PLoS One. 2007 Apr 4;2(4):e357. doi: 10.1371/journal.pone.0000357 (PMC1831490; doi:10.1371/journal.pone.0000357)
Supplement: Table S2 — NUCLEAR ALLELES DISTRIBUTION. Genotype distribution of the 4 introns (S7; α-Trop; Rag; Tpi) as a function of the population sampled (reference populations and hybrid zone populations). (0.14 MB DOC) [file pone.0000357.s014.doc]

Table S2:
