# Supplementary material for: Novelties in Hybrid Zones: Crossroads between Population Genomic and Ecological Approaches
Source: PLoS One. 2007 Apr 4;2(4):e357. doi: 10.1371/journal.pone.0000357 (PMC1831490; doi:10.1371/journal.pone.0000357)
Supplement: Table S3 — CROSS VALIDATION PROCEDURE FOR MORPHOLOGICAL CHARACTERS. C.n.n = C.n.nasus in reference zones; C.t.t = C.t. toxosotoma in reference zones. -1 = underrepresented combinations; 0 = combinations in expected proportion; 1 = overrepresented combinations; H5 = C.n.nasus from hybrid zone; T5 = C.t. toxosotoma from hybrid zone; H4T; Hi4; T4i = hybrid combinations (cf text for more explanations). (0.11 MB DOC) [file pone.0000357.s015.doc]

Table S3:

| Cross validation procedure for meristic characters | | | | | | | | | | |  | |  | |  | |  | |  |
| --- | --- | --- | --- | --- | --- | --- | --- | --- | --- | --- | --- | --- | --- | --- | --- | --- | --- | --- | --- |
|  |  |  |  |  |  |  |  |  |  |  | |  | |  | |  | |  | |
|  |  |  |  |  |  |  |  |  |  |  | |  | |  | |  | |  | |

| Cross validation procedure for plastic characters |  |  |  |  |  |
| --- | --- | --- | --- | --- | --- |
