# Supplementary material for: Novelties in Hybrid Zones: Crossroads between Population Genomic and Ecological Approaches
Source: PLoS One. 2007 Apr 4;2(4):e357. doi: 10.1371/journal.pone.0000357 (PMC1831490; doi:10.1371/journal.pone.0000357)
Supplement: Table S4 — MANCOVA ON MORPHOLOGICAL DATA SET. Order-3 analysis. Cond: Coefficient of condition, Gen. Group: Genomic group (cf. text for more details). (0.13 MB DOC) [file pone.0000357.s016.doc]

Table S4:

| MERISTIC |  |  |  |  |  |  |  |
| --- | --- | --- | --- | --- | --- | --- | --- |
|  | df | W | n^2^ | F | n.df | d.df | P |
| Cond | 1 | 1 | 0 | 0.002 | 4 | 1145 | 1 |
| Weight | 1 | 1 | 0 | 0.001 | 4 | 1145 | 1 |
| Centroid | 1 | 0.997 | 0.003 | 0.98 | 4 | 1145 | 0.418 |
| Age | 2 | 0.985 | 0.015 | 2.21 | 8 | 2288 | 0.024 |
| Year | 1 | 0.875 | 0.125 | 40.886 | 4 | 1145 | 0 |
| Gen. group | 7 | 0.707 | 0.293 | 14.822 | 28 | 4108.145 | 0 |
| Station | 3 | 0.975 | 0.025 | 2.376 | 12 | 3024.385 | 0.005 |
| Sex | 2 | 0.984 | 0.016 | 2.272 | 8 | 2288 | 0.02 |
| Age*Sex | 4 | 0.994 | 0.006 | 0.405 | 16 | 3477.285 | 0.982 |
| Age*Year | 2 | 0.997 | 0.003 | 0.458 | 8 | 2282 | 0.886 |
| Gen. group*Sex | 14 | 0.948 | 0.052 | 1.086 | 56 | 4370.403 | 0.308 |
| Gen. group*Year | 7 | 0.972 | 0.028 | 1.134 | 28 | 4079.301 | 0.286 |
| Age*Station | 6 | 0.978 | 0.022 | 1.032 | 24 | 3960.752 | 0.419 |
| Station*Sex | 6 | 0.987 | 0.013 | 0.597 | 24 | 3960.752 | 0.938 |
| Year*Sex | 2 | 0.991 | 0.009 | 1.329 | 8 | 2282 | 0.224 |
| Year*Station | 3 | 0.988 | 0.022 | 1.154 | 12 | 3013.802 | 0.311 |
|  |  |  |  |  |  |  |  |
|  |  |  |  |  |  |  |  |
|  |  |  |  |  |  |  |  |
| PLASTIC |  |  |  |  |  |  |  |
|  | df | W | n^2^ | F | n.df | d.df | P |
| Cond | 1 | 0.999 | 0.001 | 0.027 | 38 | 1148 | 1 |
| Weight | 1 | 0.999 | 0.001 | 0.026 | 38 | 1148 | 1 |
| Centroid | 1 | 0.894 | 0.106 | 3.574 | 38 | 1148 | 0 |
| Age | 2 | 0.861 | 0.138 | 2.333 | 76 | 2294 | 0 |
| Year | 1 | 0.540 | 0.459 | 25.655 | 38 | 1148 | 0 |
| Gen. group | 7 | 0.502 | 0.497 | 3.114 | 266 | 7887.5 | 0 |
| Station | 3 | 0.579 | 0.420 | 6.008 | 114 | 3433.6 | 0 |
| Sex | 2 | 0.690 | 0.309 | 6.152 | 76 | 2294 | 0 |
| Age*Sex | 4 | 0.871 | 0.129 | 1.054 | 152 | 4549 | 0.309 |
| Age*Year | 2 | 0.927 | 0.072 | 1.146 | 76 | 2288 | 0.184 |
| Gen. group*Sex | 14 | 0.616 | 0.383 | 1.046 | 532 | 14871.9 | 0.224 |
| Gen. group*Year | 7 | 0.765 | 0.234 | 1.166 | 266 | 7832.3 | 0.034 |
| Age*Station | 6 | 0.740 | 0.259 | 1.540 | 228 | 6764.3 | 0 |
| Station*Sex | 6 | 0.788 | 0.211 | 1.215 | 228 | 6764.3 | 0.016 |
| Year*Sex | 2 | 0.913 | 0.086 | 1.397 | 76 | 2288 | 0.014 |
| Year*Station | 3 | 0.679 | 0.320 | 4.130 | 114 | 3421.7 | 0 |
