# Supplementary material for: Novelties in Hybrid Zones: Crossroads between Population Genomic and Ecological Approaches
Source: PLoS One. 2007 Apr 4;2(4):e357. doi: 10.1371/journal.pone.0000357 (PMC1831490; doi:10.1371/journal.pone.0000357)
Supplement: Table S5 — ANOVA ON COEFFICIENT OF CONDITION DATA SET. (0.03 MB DOC) [file pone.0000357.s017.doc]

Table S5:

|  | df | Order-3 Sum of Sq. | Mean Sq. | F value | p(F) |
| --- | --- | --- | --- | --- | --- |
| Age | 2 | 27.982 | 13.990 | 6.923 | 0.00103 |
| Genomic class | 7 | 225.557 | 32.222 | 15.946 | <10-9 |
| Age* Genomic class | 14 | 163.407 | 11.671 | 5.776 | <10-9 |
| Residuals | 905 | 1828.737 | 2.020 |  |  |
