# Supplementary material for: Novelties in Hybrid Zones: Crossroads between Population Genomic and Ecological Approaches
Source: PLoS One. 2007 Apr 4;2(4):e357. doi: 10.1371/journal.pone.0000357 (PMC1831490; doi:10.1371/journal.pone.0000357)
Supplement: Table S6 — AGE DISTRIBUTION. Distribution of hybrid zone combinations as a function of genome dilution and age (data are in percentages). (0.06 MB DOC) [file pone.0000357.s018.doc]

Table S6:

|  |  | Age (years old) | | | | | | | | | |
| --- | --- | --- | --- | --- | --- | --- | --- | --- | --- | --- | --- |
| Dilution | | 1 | 2 | 3 | 4 | 5 | 6 | 7 | 8 | 9 | 10 |
| T5 | 0 | 6 | 42 | 29 | 13 | 8 | 3 | 0 | 0 | 0 | 0 |
|  | 1 | 16 | 32 | 30 | 11 | 8 | 3 | 0 | 0 | 0 | 0 |
|  | 2 | 10 | 32 | 26 | 29 | 3 | 0 | 0 | 0 | 0 | 0 |
|  | 3 | 5 | 43 | 24 | 14 | 5 | 5 | 5 | 0 | 0 | 0 |
|  | 4 | 3 | 32 | 34 | 21 | 11 | 0 | 0 | 0 | 0 | 0 |
|  | 5 | 4 | 14 | 31 | 35 | 14 | 2 | 0 | 0 | 0 | 0 |
|  | 6 | 0 | 26 | 47 | 21 | 5 | 0 | 0 | 0 | 0 | 0 |
|  | 7 | 2 | 15 | 30 | 39 | 9 | 4 | 2 | 0 | 0 | 0 |
|  | 8 | 4 | 17 | 48 | 30 | 0 | 0 | 0 | 0 | 0 | 0 |
| H5 | 9 | 10 | 22 | 17 | 29 | 14 | 5 | 1 | 0 | 1 | 1 |
