# Supplementary material for: Novelties in Hybrid Zones: Crossroads between Population Genomic and Ecological Approaches
Source: PLoS One. 2007 Apr 4;2(4):e357. doi: 10.1371/journal.pone.0000357 (PMC1831490; doi:10.1371/journal.pone.0000357)
Supplement: Table S7 — SEX RATIO IN THE HYBRID ZONE. References did not indicate a sex ratio significantly different from 1 for the two species studied in allopatric populations [1], [2]. F = female, M = male and I = indeterminate. 1) Gozlan RE (1998) Environmental biology and morphodynamics of the sofie Chondrostoma toxostoma (Cyprinidae), with emphasis on early development. PhD, Université Toulouse, France. 196p. 2) Nelva-Pasqual A (1985) Biogéographie, démographie et écologie de Chondrostoma nasus nasus (L., 1758) Hotu (Poisson, Téléostéen, Cyprinidé). PhD Université Claude-Bernard-Lyon I. 340p. (1.51 MB DOC) [file pone.0000357.s019.doc]

Table S7:
