# Supplementary material for: Novelties in Hybrid Zones: Crossroads between Population Genomic and Ecological Approaches
Source: PLoS One. 2007 Apr 4;2(4):e357. doi: 10.1371/journal.pone.0000357 (PMC1831490; doi:10.1371/journal.pone.0000357)
Supplement: Table S8 — VARIABLES INTERACTIONS. A) order-two interactions (chi2); B) order-three interactions (log-linear model) and main associated results. (0.12 MB DOC) [file pone.0000357.s020.doc]

Table S8:

A)

| **Tested interactions on specimens distribution** | **chi2 results** | **Principal associations** |
| --- | --- | --- |
|  |  |  |
|  |  |  |
| **Genetic class X Station** | Chi obs.= 568.1858 pvalue<10^-5^ | H5 asso+ with Ca |
|  |  | H5 asso- with Pe |
|  |  | T5 asso+ with Pe |
|  |  | T5 asso- with Ca |
|  |  | H5 asso- with Ma |
|  |  | H5 asso+ with Bu |
|  |  | T5 asso- with Bu |
|  |  | T4i asso+ with Ma |
|  |  | H4T asso+ with Bu |
|  |  |  |
| **Genetic class X Year** | Chi obs.=62.7736 pvalue<10^-5^ | T4i asso+ with 2001 |
|  |  | T4i asso- with 2002 |
|  |  |  |
| **Genetic class X Age** | Chi obs.=179.5623 pvalue<10^-5^ | H4T asso+ with 4 years old |
|  |  | T5 asso- with 4 years old |
|  |  | Hi4 asso+ with 4 years old |
|  |  | T4i asso+ with 1 year old |
|  |  | T5 asso+ with 2 years old |
|  |  | H5 asso+ with 4 years old |
|  |  |  |
| **Genetic class X Sex** | Chi obs.=21.1943 pvalue>0.10 | Not significant effect |
|  |  |  |
|  |  |  |
| **Sex X Station** | Chi obs.=128.6324 pvalue<10^-5^ | Immature asso+ with Ma |
|  |  | Male asso- with Ma |
|  |  | Male asso+ with Pe |
|  |  | Femelle asso+ with Bu |
|  |  |  |
| **Year X Station** | Chi obs.=10.8229 pvalue>0.01 | Not significant effect |
|  |  |  |
|  |  |  |
| **Age X Station** | Chi obs.=424.6809 pvalue<10^-5^ | 1 year old asso+ with Ca |
|  |  | 4-5 years old asso+ with Bu |
|  |  | 2 years old asso+ with Pe |
|  |  | 2 years old asso+ with Ma |
|  |  | 6 years old asso+ with Ca |
|  |  |  |
| **Sex X Age** | Chi obs.=162.3327 pvalue<10^-5^ | Immatures asso+ with 1 year old |
|  |  | Male asso+ with 1 year old |
|  |  |  |
| **Year X Age** | Chi obs.=92.5760 pvalue<10^-5^ | 5 years old asso- with 2001 |
|  |  | 5 years old asso+ with 2002 |
|  |  |  |
| **Sex X Year** | Chi obs.=152.6815 pvalue<10^-5^ | Immatures asso- with 2001 |
|  |  | Immatures asso+ with 2002 |
|  |  | Male asso+ with 2001 |
|  |  | Male asso- with 2002 |

| **Order-3 interactions (dev=72.134; p=5.10-10)**  B) | **chi^2^ results** | **Principal associations** |
| --- | --- | --- |
|  |  |  |
|  |  |  |
| **Genetic class X Station x 2001** | Chi obs.= 276.8705 pvalue<10^-5^ | H5 asso+ with Ca |
|  |  | T4i asso+ with Ma |
|  |  | T5 asso+ with Pe |
|  |  | H5 asso+ with Bu |
|  |  | T5 asso- with Bu |
|  |  | H4T asso+ with Bu |
|  |  |  |
| **Genetic class X Station x 2002** | Chi obs.= 342.8434 pvalue<10^-5^ | H5 asso+ with Ca |
|  |  | H5 asso- with Pe |
|  |  | T5 asso- with Ca |
|  |  | T5 asso+ with Pe |
|  |  | H5 asso- with Ma |
|  |  | H5 asso+ with Bu |
